# Identification, Isolation and Structural Elucidation of New Inner Salt Impurity in Vildagliptin and Metformin Tablet by Using Supercritical Fluid Chromatography, NMR, HRMS

**DOI:** 10.1002/jssc.70218

**Published:** 2025-07-12

**Authors:** Saravana Kumar Meganathan, Bijaya Ketan Sahoo, Y. Rajender Reddy, K. Rajani, Peedi Rajasekhara Reddy

**Affiliations:** ^1^ Department of Chemistry GITAM School of Science GITAM Deemed to be University Hyderabad India; ^2^ Natco Research Centre Natco Pharma Ltd. Industrial Estate Hyderabad India

**Keywords:** high resolution mass spectrometry, impurity, isolation, NMR, SFC, vildagliptin and metformin

## Abstract

Vildagliptin is an orally active antihyperglycemic agent used for the treatment of type 2 diabetes mellitus in adults as monotherapy either alone or in combination with metformin. The presence of impurities in drug substances or drug products could be toxic and unsafe (injurious) to the user, and it should be ensured that drugs must be free of or controlled from any impurities to the specified level as per International Council for Harmonization guidelines before that reaches the user. That is the reason why work on identification, isolation, and characterization of impurities in drug substances and drug products is very important towards ensuring drug safety and efficacy. Further, product‐related impurity isolation and characterization is an important aspect of pharmaceutical processes. The aim of the work is to identify a new impurity which was detected in vildagliptin and metformin tablets under stability storage conditions by using high‐performance liquid chromatography (HPLC). The new impurity was isolated and characterized by using comprehensive analysis of HPLC, flash chromatography, supercritical fluid chromatography, Fourier transform infrared spectroscopy, high‐resolution mass spectrometry, 1D‐NMR [^1^H and ^13^C], and 2D‐NMR (^1^H‐^1^H correlated spectroscopy, total correlated spectroscopy, ^1^H‐^13^C heteronuclear single quantum coherence spectroscopy, ^1^H‐^13^C and ^1^H‐^15^N heteronuclear multiple bond coherence spectroscopy) spectroscopic data.

## 1 Introduction

**FIGURE 1:**
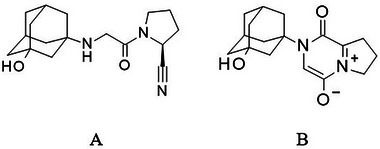
Structures of (A) vildagliptin, (B) unknown impurity.

Diabetes is a chronic and metabolic disease that is characterized by an elevated blood sugar level. Type 2 diabetes, the most common among adults, is known to occur when the body is resistant to insulin or the body doesn't make enough insulin, which is the hormone that makes the body use sugar and plays a role in regulating the blood sugar levels. There are several drugs available commercially to control sugar levels and thus in treating type‐2 diabetes. Glucagon‐like peptide 1 (GLP‐1) is the hormone that is secreted by the intestinal L‐cells that is known to stimulate the secretion of insulin. But the enzyme dipeptidyl peptidase 4 (DPP‐4) inactivates GLP‐1, whose impaired secretion is linked to diabetes. Vildagliptin (VG), as shown in Figure 1A is a type 2 diabetes medication being used in controlling blood sugar by selective inhibition of DPP‐4. Further, VG prevents the breakdown of glucose‐dependent insulinotropic polypeptide (GIP) by DPP‐4. Thus, prevention of the breakdown of GIP, which is an incretin hormone being secreted from the small intestine in response to the nutrients improves the glycemic control. The risk of hypoglycemia (low blood sugar) is relatively low in clinical trials associated with VG [1, 2]. European Medicines Agency in the year 2008 approved the use of oral VG for the treatment of type 2 diabetes, either alone or in combination with metformin (MF), sulfonylureas, or thiazolidinediones in patients who could not achieve adequate glycemic control with monotherapy [3].

Comprehensive insight into the chemical stability of VG using chromatography and spectroscopy methods has been reported [4]. Process and degradation products of VG have been reported [5, 6]. Validated high‐performance liquid chromatography (HPLC) methods for the estimation of VG and VG in combination with MF in pharmaceutical dosage forms [7, 8] and assays for the estimation of VG and MF using reverse‐phase high‐performance liquid chromatography (RP‐HPLC) [9] have been reported. Ultra‐performance liquid chromatography (UPLC/HPLC) MS determination of VG and its organic impurities, degradation products and genotoxic impurities have been reported [10, 11, 12, 13]. The development and validation of the HPLC method using the quality by design (QbD) approach and its application to forced degradation studies has been reported [14]. The synthesis of VG impurities has been disclosed in VG process details [15, 16]. Forced degradation studies of VG raw material alone and in the presence of excipients using HPLC‐UV analysis have been studied [17]. In the pharmaceutical industry, it is essential that the process related, or degradation impurities formed in drug substances (as per the ICH guideline Q3A (R2)) [18] or in drug products (as per the ICH guideline Q3B (R2)) [19] must be identified since they have a significant impact on the quality and safety of the drug substance and drug product. Therefore, impurity profiling studies, even at the level below 0.05% in drug substance and drug product have gained attention, and hence it is important to elucidate the structure of those impurities [20, 21, 22, 23]. The forced degradation and stability studies of pharmaceutical products have been well reported [24, 25, 26, 27, 28]. In addition to the reported process and degradation related impurities, a new unknown impurity (Figure 1B) was observed during accelerated stability storage conditions. To the best of our knowledge, this new unknown impurity has not been reported in the VG or VG and MF process or degradation. In this context, a comprehensive study has been undertaken to identify, isolate, and characterize the unknown impurity. Supercritical fluid chromatography (SFC) was used for the isolation of impurity, which has more advantages over liquid chromatography. SFC is considered as a greener approach due to less solvent consumption that is more expensive, toxic, and harmful to the environment. In recent days, the application of SFC has been used and succeeded in various industries for analytical and preparative applications [29, 30, 31, 32, 33, 34]. SFC is preferable complementary with conventional preparative HPLC and many research articles have been published using SFC [35, 36, 37]. In a preparative application, the use of SFC increases the chiral and achiral purification due to its high diffusivity and low viscosity, which allows for higher flow rates (even at lower particle size stationary phases), improved efficiency, and support for orthogonal selectivity. Also, preparative SFC provide the benefits of easy solvent removal after purification, reduced solvent consumption, reduced cost, increased productivity, and requires less use of ancillary equipment like pH meters, sonicators, and freeze dryers.

## 2 Materials and Methods

### 2.1 Chemicals and Reagents

VG and MF tablets and API were received from NATCO formulations division, Kothur, Hyderabad, India. VG known impurities (monoketo impurity, diketo impurity, and amide impurity) were prepared by NATCO Research Centre, Hyderabad, India. The solvents acetonitrile and methanol (HPLC grade) were procured from J.T. Baker. Ethyl acetate, dichloromethane, sodium dihydrogen phosphate, ammonium acetate (chromatographic grade), orthophosphoric acid, and ammonia solution were procured from Merck. The pure carbon dioxide with dip tube was procured from Spec Gases and Equipments with purity of 99.99%. NMR solvent CDCl_3_ was procured from Cambridge Isotope Laboratories, USA. The water used for the buffer preparation was from the Milli‐Q water system.

### 2.2 Methodology

#### 2.2.1 Analytical HPLC

Waters Alliance e2695 separation module equipped with a photodiode array (PDA) detector was used for analysis, and Empower software was used for data acquisition and data processing. The chromatographic separation was achieved by using an X‐Bridge, C18 (4.6 × 250 mm) column, where 5 µm column was employed for the separation using gradient mode. The gradient program was time (min)/% B (mobile phase): 0/7, 25/7, 35/16, 45/16, 45/24, 65/50, 80/50, 85/7, and 95/7. The column oven temperature was maintained at 50°C. The buffer consisted of 1.14 g of Na_2_HPO_4_ in a liter of Milli‐Q water with setting the pH to 7.5 using orthophosphoric acid. Mobile phase A consisted of a mixture of buffer and acetonitrile in the ratio of 98:2 (v/v), and mobile phase B consisted of a mixture of buffer and acetonitrile in the ratio of 30:70 (v/v). The flow rate was maintained at 0.8 mL/min with an injection volume of 15 µL, and the UV detection was monitored at 210 nm. Diluted HCl (0.02 N) in water was used as a diluent.

#### 2.2.2 Liquid Chromatography Mass Spectrometry (LC‐MS)

Waters Xevo TQ‐XS Triple Quadrupole Mass Spectrometer coupled with UPLC was used for the analysis and MassLynx software was used for data acquisition and data processing. The chromatographic separation was achieved by using an X‐Bridge, C18 (4.6 × 250 mm) column, where 5 µm column was employed for the separation using gradient mode. The gradient program was time (min) / % B: 0/7, 25/7, 35/16, 45/16, 45/24, 65/50, 80/50, 85/7, and 95/7. The column oven temperature was maintained at 50°C. The buffer consisted of 10 mM ammonium acetate in Milli‐Q water and was adjusted to the pH 8.5 with ammonia solution. The mobile phase A consisted of a mixture of buffer and acetonitrile in the ratio of 98:2 (v/v), and the mobile phase B consisted of a mixture of buffer and acetonitrile in the ratio of 30:70 (v/v). The flow rate was maintained at 0.8 mL/min with an injection volume of 15 µL and the UV detection was monitored at 210 nm. A mixture of acetonitrile and water in the ratio of 70:30 (v/v) was used as a diluent. The MS analysis was performed using the electrospray ionization (ESI) source in positive mode. The instrument parameters consisted of setting the capillary and cone voltage at 3000 and 20 V respectively, cone and desolvation gas flow at 150 and 1000L/h, respectively; and the temperature of the source and desolvation at 150°C and 500°C, respectively.

#### 2.2.3 Liquid Chromatography High Resolution Mass Spectrometry (LC‐HRMS)

The Waters Synapt G2‐Si high definition mass spectrometry (HDMS) was used for the LC‐HRMS analysis, and MassLynx software was used for data acquisition and data processing. The following chromatographic conditions were used for the analysis. Union was used in place of column using isocratic mode with mobile phase A and mobile phase B in the ratio of 10:90 (v/v). The column oven temperature was maintained at 25°C. The mobile phase A consisted of 10 mm ammonium formate with 0.1% formic acid in Milli‐Q water, and the mobile phase B consisted of acetonitrile. The flow rate was maintained at 0.1 mL/min with an injection volume of 2 µL, and the UV detection was monitored at 210 nm. A mixture of acetonitrile and water in the ratio of 70:30 (v/v) was used as a diluent. The MS analysis was performed using the electrospray ionization (ESI) source in positive ion mode. The instrument parameters consisted of setting the analyzer mode as sensitivity, the capillary and cone voltage at 2.0 kV and 20 V, respectively, cone and desolvation gas flow as 50 and 650 L/h respectively, and the temperature of the source and desolvation at 100°C and 250°C, respectively.

#### 2.2.4 Flash Chromatography System (Flash)

The pre‐purification was performed using a Gilson preparative liquid chromatography (PLC) 2500 cum flash chromatography system equipped with a UV dual wavelength detector and controlled by Glider software. The conditions employed for the isolation of impurity is as follows. A 100 g flash cartridge packed with Chromatorex Diol 10/30μ resin was used for the separation. The gradient program consisted of time (min) / % B: 0/0, 5/0, 10/10, 15/10, 20/16, 25/18, 30/20, 45/20. Solvent A consisted of ethyl acetate, and solvent B consisted of methanol. The flow rate was maintained at 75 mL/min, and the UV detection was monitored at 210 and 375 nm (*λ*_max_ of the unknown impurity). The sample was loaded using a solid loader coated with diol and silica in 1:3 ratio w/w (dry sample mode).

#### 2.2.5 Supercritical Fluid Chromatography (SFC)

The second purification was performed by using Waters SFC 150 mgm equipped with a photodiode array (PDA) detector. Chromscope software was used for instrument control and data acquisition. The chromatographic conditions employed for the isolation of unknown impurity consisted of a Torus‐2‐PIC (100 × 19) mm column where 5 µm column was used for the separation in isocratic mode using phase A and phase B in the ratio of 90:10 v/v. Phase A was CO_2_ and phase B was a mixture of acetonitrile and methanol in the ratio of 70:30 (v/v). The flow rate was maintained at 60 mL/min with an injection volume of 2 mL for each injection, and the UV detection was monitored at 210 and 375 nm (*λ*_max_ of the unknown impurity). The back pressure was set at 2000 psi. The sample was prepared at 50 mg/mL using methanol.

#### 2.2.6 Nuclear Magnetic Resonance (NMR)

The NMR experiments were performed using a Bruker Avance Neo 600 MHz NMR spectrometer equipped with 5 mm (BBFO) probe in CDCl_3_ as solvent at 25°C. ^1^H NMR measurements were carried out at 600 MHz, while ^13^C NMR and ^15^N NMR experiments were performed at 150 and 60 MHz respectively. The proton and carbon chemical shifts were reported using the *δ* scale in ppm. Tetramethylsilane (TMS) (*δ* = 0.00 ppm) and CDC1_3_ (*δ* = 77.00 ppm) were the internal standards in ^1^H and ^13^C NMR spectra, respectively. The standard pulse sequences and program provided by Bruker were used for 1D and 2D NMR data. TopSpin (ver: 4.3) software was used for data processing.

#### 2.2.7 Fourier Transform Infrared Spectroscopy

FT‐IR data was recorded on a Bruker FT‐IR Spectrometer (Model: Alpha) in KBr medium.

## 3 Results and Discussion

### 3.1 Identification of Unknown Impurity

**FIGURE 2:**
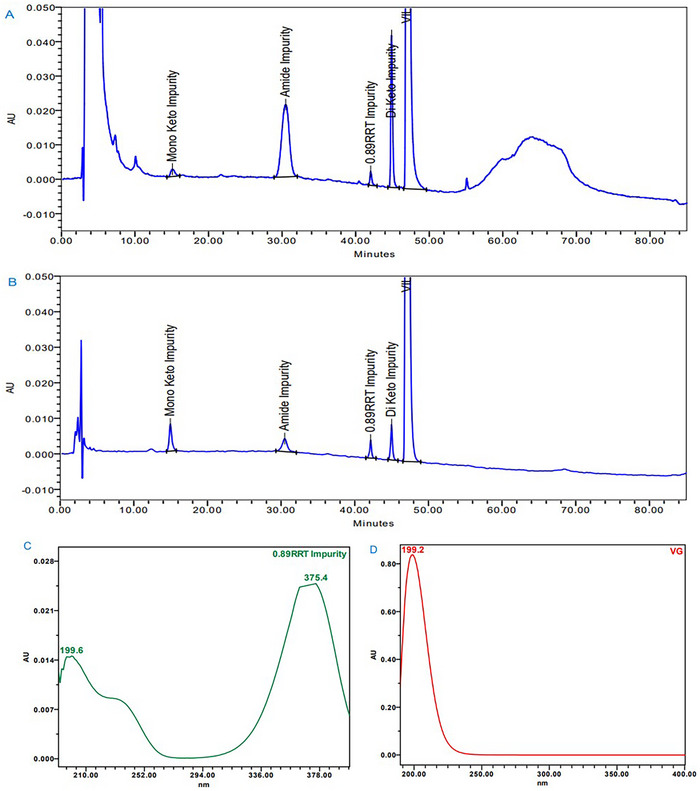
(A) HPLC chromatogram of vildagliptin and metformin tablet sample (40°C/75%RH, 6 months), (B) HPLC chromatogram of vildagliptin spiked with unknown impurity and other known impurities, (C) UV spectrum of unknown impurity and (D) UV spectrum of vildaglitpin.

The VG and MF combination tablets were exposed and evaluated under accelerated stability storage conditions at 40°C/75% RH as per ICH guideline Q1A(R2) [38]. The VG and MF tablet powder, which was equivalent to 100 mg of VG in 100 mL volumetric flask was weighed and dissolved in analytical diluent by orbital shaker followed by centrifuged and filtered. Then the sample solution was analyzed on analytical HPLC using LC chromatographic conditions as mentioned in Section 2.2.1. An unknown impurity (UI) was observed at 0.3% level (Figure 2A), which was eluted at RRT 0.89 with respect to VG in tablets stored at accelerated storage conditions (40°C/75% RH, 6 months).

After the preliminary mass data of the impurity obtained from the LC‐MS/MS study, it was identified that the unknown impurity was related to VG based on the mass fragmentation pattern as given in Table 1. The UV spectrum of the unknown impurity (UI) exhibited a bathochromic shift with *λ*_max_ at 375 nm compared to VG at 200 nm, as in Figure 2D, and indicates the formation of quaternization.

**TABLE 1:** MS and MS/MS fragmentation of vildagliptin and impurities.

#	RRT	% Area	Observed mass (*m/z*)	Fragments (MS/MS, *m/z*)	Name
1	0.89	0.33	303.0	153.1, 132.7, 110.9, 106.9, 93.2	UI
2	0.95	1.07	305.1	154.9, 151.4, 133.4, 126.9	DKI
3	1.00	95.80	304.2	154.0, 151.0, 133.1, 106.9, 96.9	VG

About 20 tablets of VG and MF were crushed and stirred in 100 mL of dichloromethane for an hour. Then, the dichloromethane layer was filtered to remove the undissolved matter and evaporated to dryness using a rotary evaporator. The sample obtained was pre‐purified by flash chromatography using conditions mentioned in Section 2.2.4. The fractions were collected based on UV absorbance at 375 nm, where the unknown impurity has maximum UV response. The unknown impurity was eluted between 24 and 27 min in the specified chromatographic condition. The unknown impurity‐enriched fractions were pooled together and evaporated to dryness using a rotary evaporator. Finally, the enriched sample obtained from flash purification was further purified by SFC using conditions mentioned in Section 2.2.5. The enriched fractions were pooled together and distilled out to dryness using a rotary evaporator to get the free solid. The isolated impurity with a purity of 95% was used for the structure characterization. The ^1^H NMR, C^13^ NMR, MS, and MS/MS data have been provided in Figures S1–S4.

### 3.2 Thermal Degradation of Vildagliptin for Enrichment of Unknown Impurity

After structure confirmation of the unknown impurity from VG and MF tablets, the thermal degradation was studied for VG API to enrich the level of unknown impurity. VG API was exposed to thermal degradation in the range of 110°C–200°C at different time intervals (30 to 60 min). At 110°C, the formation of impurity was less and observed at 0.8% level after 60 min. At 150°C, the formation of impurity was higher at 0.8% and 2.0% levels for 30 and 60 min, respectively. Further continuation led to the formation of other impurities. At 200°C, the product was completely charred and led to the formation of unwanted impurities and was not useful. Finally, the VG API was exposed to thermal degradation using an optimized condition of 150°C for 60 min, where the unknown impurity was observed at 2.0% level. The thermal degradation process was reproducible for the formation of unknown impurity. The LC‐MS analysis was performed to confirm the formation of impurity in the thermal degradation sample using the condition mentioned in Section 2.2.2 and observed protonated molecules at *m/z* 303.1.

### 3.3 Isolation of Unknown Impurity

The VG API sample (5 g) was exposed to thermal degradation at 150°C for 60 min and analyzed on HPLC using the conditions mentioned in Section 2.2.1 to confirm the formation of impurity. Thereafter, the degraded sample (5 g) was dissolved in 50 mL of dichloromethane and coated on diol silica (15 g) in 1:3 ratios using a rotary evaporator. The coated sample was packed in the solid loader assembly and purified on 100 g flash cartridge (Chromatorex Diol, 10/30 µm) using the flash chromatographic conditions as mentioned in Section 2.2.4. The fractions were collected based on UV absorbance at 375 nm, where the unknown impurity exhibited the maximum UV response. The unknown impurity was eluted between 24 and 27 min in the specified chromatographic condition. The enriched fractions were pooled together and evaporated to dryness using a rotary evaporator. About 0.5 g of unknown impurity with a purity of 20%–30% was obtained after flash purification. Thereafter, the enriched sample (0.5 g, 20%–30%) was further purified on an SFC column (Torus‐2‐PIC (100 × 19) mm, 5 µm) using SFC chromatographic conditions as mentioned in Section 2.2.5. The enriched sample was dissolved in methanol at 50 mg/mL concentration, and injected 2 mL (100 mg) of sample solution to the SFC column. The fractions were collected based on UV absorbance at 375 nm. The enriched fractions with a purity of about 95% were pooled together and evaporated to dryness using a rotary evaporator to get a free flowing solid. The isolation process was repeated multiple times with the VG thermal degradation sample (5 × 10g) and confirmed that the process was reproducible. Finally, about 500 mg of unknown impurity was isolated with a purity of 94.28% and characterized by IR, UV, HRMS, and NMR (1D and 2D) spectroscopic data.

### 3.4 Structural Elucidation of Unknown Impurity

**FIGURE 3:**
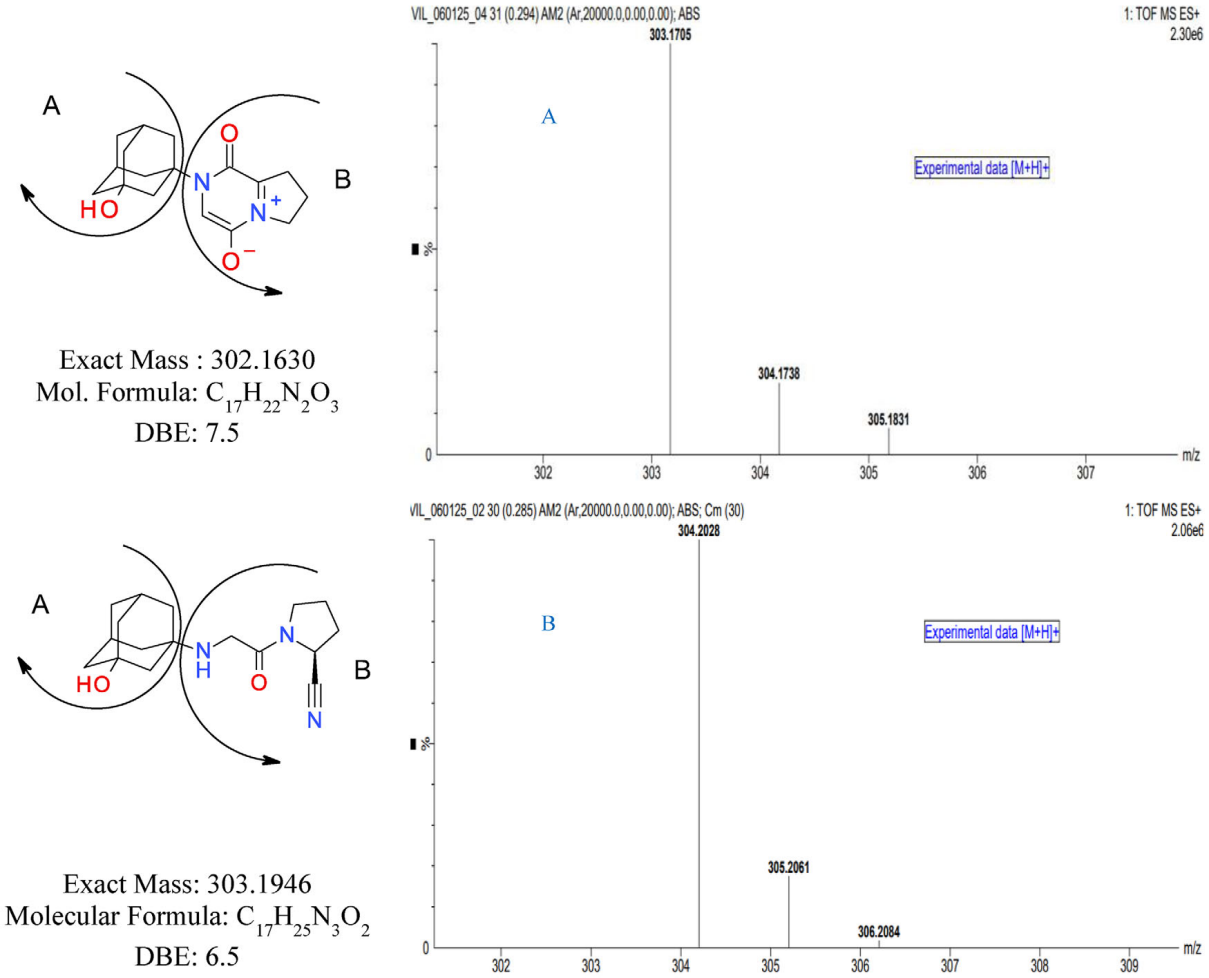
Mass spectrum of (A) Unknown impurity and (B) Vildagliptin.

The structure of UI has been divided into part A (2‐adamantanol) and part B, as shown in Figure 3 to get a better explanation of structure elucidation. The comparison of the FT‐IR spectral features (Figures S5 and S6) and the data (Table 2) of unknown impurity with VG showed the absence of the characteristic signals related to C≡N and N─H though the hydroxyl stretching was consistent in both the spectra. Thus, this observation indicates that there is a change in part B.

**TABLE 2:** FT‐IR assignments of vildagliptin and unknown impurity.

Frequency(cm^−1^)	Assignments		Mode of vibration
	Vildagliptin	Unknown impurity	
O─H	3377 (broad)	3385	Stretching
>N─H	3294	Absent	Stretching
Aliphatic C─H	2914, 2932	2920, 2854	Stretching
C≡N	2237	Absent	Stretching
C═O	1657	1616	Stretching
O─H	1355	1355	Bending
C─O	1120	1125	Stretching

The HR‐MS data of the unknown impurity showed the protonated molecule at *m/z* 303.1705, [M+H]^+^ as shown in Figure 3A. The observed *m/z* suggests that the UI has one amu less compared to the *m/z* of VG, as shown in Figure 3B.

The elemental composition of the protonated UI was determined as C_17_H_23_N_2_O_3_ and thus indicates its molecular formula to be C_17_H_22_N_2_O_3_. The comparison of the unknown impurity elemental composition with VG (C_17_H_25_N_3_O_2_) shows that the impurity has one additional single oxygen atom with the deletion of three hydrogen and one nitrogen atoms. The MS/MS data of the UI and VG showed the common fragments at *m*/*z* 132.7 and 106.9, suggesting the intact of 2‐adamantanol moiety in UI. The 1D NMR spectral features of UI (Figures S7 and S8) compared to VG (Figures S9 and S10) and the NMR data assignment (Table 3) showed the presence of 2‐adamantanol moiety (part A) in the UI without any change.

**TABLE 3:** ^1^H and ^13^C NMR assignments of vildagliptin, unknown impurity and diketo impurity.

#	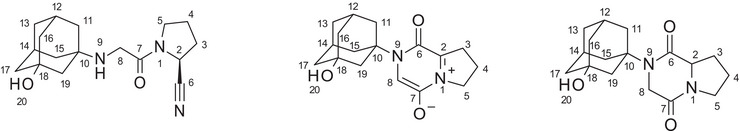								
Position	1H *δ* (ppm)	Multiplicity (J in Hz)	13C *δ* (ppm)	1H *δ* (ppm)	Multiplicity (J, Hz)	13C *δ* (ppm)	1H *δ* (ppm)	Multiplicity (J, Hz)	13C *δ* (ppm)
2	4.77–4.78	(dd,1H) (7.6,4.0)	46.49	—	—	176.72	3.99–4.02	t, (7.8)	60.45
3	2.23–2.39	(m, 2H)	29.86	2.66–2.68	(t, 2H) (6.6)	38.17	1.99–2.03	m, 1H	28.73
							1.88–1.92	m, 1H	
4	2.10–2.19	(m, 2H)	25.00	2.16–2.29	(m, 2H)	21.46		m, 2H	22.86
5 Ha	3.52–3.63	(m, 1H)	45.42	4.02–4.04	(t,2H) (6.0)	39.99	3.52–3.59	m, 2H	44.96
5 Hb	3.39–3.50	(m, 1H)							
6	—	—	118.16	—	—	154.17	—	—	168.55
7	—	—	170.61	—	—	122.29	—	—	164.07
8	3.39–3.50	(m, 2H)	43.41	6.65	(s, 1H)	106.94	4.08–4.11	(d,1H) (15.6)	47.33
							3.85–3.88	(d, 1H) (16.2)	
9 (NH)	1.82	(br, 1H)	—	—	—		—	—	
10	—	—	53.40	—	—	64.80	—	—	61.45
11	1.54–1.69	(m, 2H)	41.32	2.16–2.29	(m, 2H)	38.93	2.08–2.19	(m, 2H)	38.35
12	2.23–2.39	(m, 1H)	30.68	2.42	(s, 1H)	30.91	2.31	(m, 1H)	30.98
13	1.54–1.69	(m, 2H)	35.10	1.58–1.72	(m, 2H)	33.97	1.51–1.59	(m, 2H)	34.71
14	2.23–2.39	(m, 1H)	30.68	2.42	(s, 1H)	30.91	2.31	(m, 1H)	30.91
15	1.54–1.69	(m, 2H)	41.15	2.16–2.29	(m, 2H)	38.93	2.08–2.19	(m, 2H)	38.35
16	1.54–1.69	(m, 2H)	44.38	1.75–1.84	(m, 2H)	43.28	1.67–1.75	(m, 2H)	43.84
17	1.54–1.69	(m, 2H)	44.36	1.75–1.84	(m, 2H)	43.28	1.67–1.75	(m, 2H)	43.87
18	—	—	69.53	—	—	69.42	—	—	69.47
19	1.54–1.69	(m, 2H)	49.88	2.16–2.29	(m, 2H)	47.93	2.08–2.19	(m, 2H)	47.90
20 (OH)	4.38 (4.40)	(s, 1H)	—	—	—	—	—	—	—

1D NMR and 2D NMR data are consistent with the IR and MS data and thus confirm the intact of the 2‐adamantanol moiety in the structure of the UI. The 2D NMR (Figures S11–S19) data of the part B of UI shows three quaternary carbons at 122.29 (C‐7), 154.17 (C‐6), and 176.72 (C‐2) ppm, one methane at 106.94 (C‐8)/6.65 (H‐8) ppm and three methylene carbons at 38.17 (C‐3)/2.66‐2.68 (H‐3), 21.46 (C‐4)/2.16‐2.29 (H‐4), and 39.99 (C5)/4.02‐4.04 (H‐5) ppm. The COSY data of VG showed that one methane (H‐2) and three methylene (H‐3,4,5) are in the same spin system. However, the COSY data of the UI (Figure S11) shows correlations between three methylene protons (H‐3,4,5) only. Thus, the absence of the aliphatic methane at position ‘2’ suggests that methane has become a quaternary carbon that is observed at 176.72 (C‐2). The methane carbon observed at 106.94 (C‐8) / 6.65 (H‐8) ppm in UI was found to be sp2 hybridized. Further, the absence of methylene and the presence of sp2 hybridized methane suggest that the methylene at position ‘8’ becomes a double bonded carbon.

The methylene signal at 4.02 (H‐5) ppm showed HMBC (Figure S14) correlation with the carbon signals at 176.72 (C‐2), 21.46 (C‐4), and 122.29 (C‐7) ppm. The methylene signal at 2.66(H‐3) ppm showed HMBC correlation with the carbon signals at 176.72 (C‐2), 21.46 (C‐4), and 154.17 (C‐6) ppm. The methane signal at *δ* 6.65 (H‐8) ppm showed HMBC correlations with the carbon signals at 154.17 (C‐6), 122.29 (C‐7), and 64.80 (C‐10) ppm. The HMBC correlations of the methane signal at 6.65 (H‐8) ppm confirm that there is a cyclization and connection between part A and part B through the nitrogen atom (N‐9). The ^15^N NMR chemical shifts were extracted from ^1^H‐^15^N HMBC data for further confirmation of the UI (Figures S15 and S16) after comparing with the data of VG (Figure S20) and diketo impurity (Figure S21) shown in Figure S22. The methane signal at 6.65 (H‐8) ppm showed HMBC correlations with two nitrogens at 174.4 (N‐1) and 209.3 (N‐9) ppm. However, the methylene signals at 2.16‐2.29 (H‐11, H‐15, and H‐19) ppm showed HMBC correlation with only one nitrogen at 209.3 (N‐9) ppm and confirms the position of methane and connection of cyclization. The ^1^H‐^15^N HMBC assignments have been shown in Table 4. In addition, the NMR data of the UI was compared with the data of VG diketo impurity (Figures S23–S26 and Tables 3, 4) and observed significant chemical shift changes of carbon at C‐2, C‐3, C‐4, C‐5, proton at H‐3, H‐4, H‐5, and nitrogen at N‐1. These chemical shift changes at the observed atoms indicate that there is an internal salt formation on part B of the UI structure. The UV data of the UI showed *λ*_max_ at 375 nm with a bathochromic shift that supports the formation of quaternization (inner salt formation) at the pyrrolidine ring with increased charge transfer [39]. Thus, based on the NMR, HRMS, IR, and UV data, the structure of the unknown impurity was identified as 2‐(3‐hydroxy‐1‐adamantyl)‐1‐oxo‐7, 8‐dihydro‐6H‐pyrrolo[1,2‐a]pyrazin‐5‐ium‐4‐olate (Figure 1B and Table 3).

**TABLE 4:** ^1^H‐^15^N HMBC NMR assignments of vildagliptin, unknown impurity and diketo impurity.

Position	Vildagliptin (VG)		Unknown impurity (UI)		Diketo impurity (DKI)	
	15N *δ* (ppm)	1H *δ* (ppm)	15N *δ* (ppm)	1H *δ* (ppm)	15N *δ* (ppm)	1H *δ* (ppm)
1	123.4	2.23–2.39 (H‐3)	174.4	6.65 (H‐8)	127.5	3.50–3.60 (H‐5), 4.08–4.11 (H‐8)
6	246.4 & 250.8	4.77–4.78 (H‐2)	—	—	—	—
9	51.8	3.39–3.50 (H‐8), 1.54–1.69(H‐11, H‐15, H‐19)	209.3	6.65 (H‐8), 2.16–2.29 (H‐11, H‐15, H‐19)	126.0	3.85–3.88 (H‐8), 2.31 (H‐12, H‐14) 2.08–2.19 (H‐11, H‐15, H‐19)

The impurity isolated from the VG & MF tablet and the impurity isolated from the thermal degradation of VG API are the same, and the analytical data are comparable and consistent. The degradation pathway of VG under basic and oxidation conditions has been reported [5]. The unknown impurity was a thermal/oxidative degradation product of VG. This impurity arises from heat or light‐induced rearrangement of the pyrrolidine ring [40, 41] present in VG. The proposed degradation pathway of the UI has been shown in Figure S27.

## 4 Conclusion

The degradation profile of VG and MF tablets was studied under stability stressed storage conditions, and a new impurity was identified, isolated, and characterized by Flash, SFC, IR, LC‐HRMS, and NMR. The structure of the impurity was confirmed by using spectral data. All the FT‐IR, UV, NMR, and HR‐MS/MS data are provided.

## Author Contributions

All the concepts, techniques, and data were generated by Saravana Kumar Meganathan. In addition, the article was drafted, updated, implemented, and edited. It was confirmed that the research was conducted collaboratively with the assistance of Bijaya Ketan Sahoo, Rajender Reddy Yerla, K Rajani, and Peedi Rajasekhara Reddy.

## Conflicts of Interest

The author declares no conflicts of interest.

## Supporting information




**Supporting file 1**: jssc70218‐sup‐0001‐figuresS1‐S27.docx
