# Supplementary material for: Identification, Isolation and Structural Elucidation of New Inner Salt Impurity in Vildagliptin and Metformin Tablet by Using Supercritical Fluid Chromatography, NMR, HRMS
Source: J Sep Sci. 2025 Jul 12;48(7):e70218. doi: 10.1002/jssc.70218 (PMC12254903; doi:10.1002/jssc.70218)
Supplement: Supplementary file 1 — Supporting file 1: jssc70218‐sup‐0001‐figuresS1‐S27.docx [file JSSC-48-e70218-s001.docx]

**SUPPLEMENTARY INFORMATION**

Identification, Isolation and Structural elucidation of new inner salt impurity in Vildagliptin and Metformin tablet by using Supercritical Fluid Chromatography, NMR, HRMS


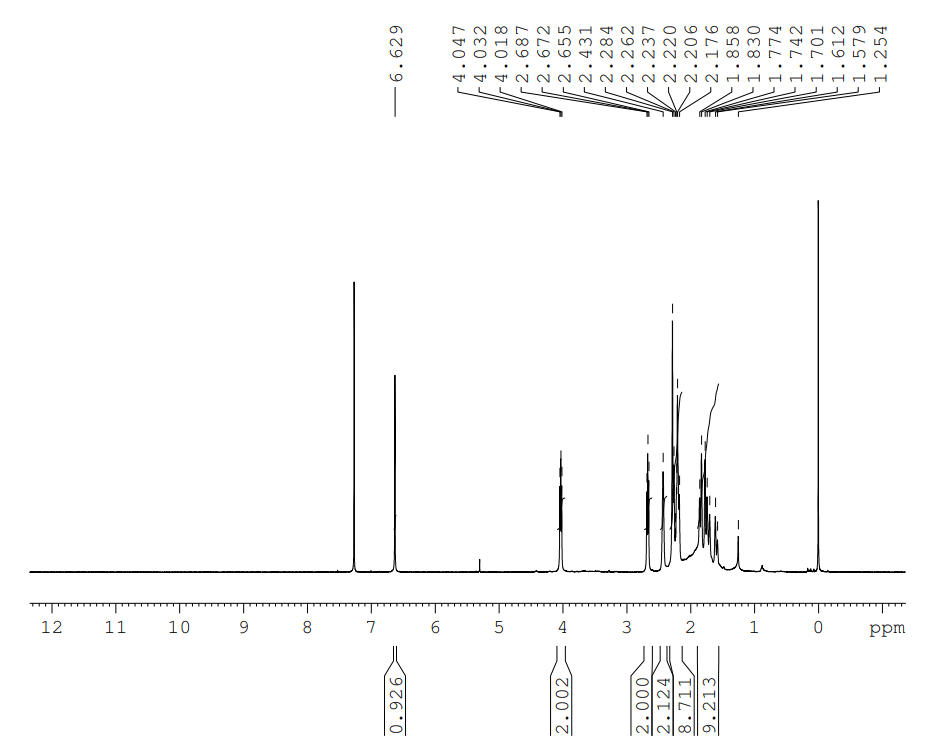


**Figure S1 |** ^1^H NMR spectrum of unknown impurity (vildagliptin and metformin tablet)


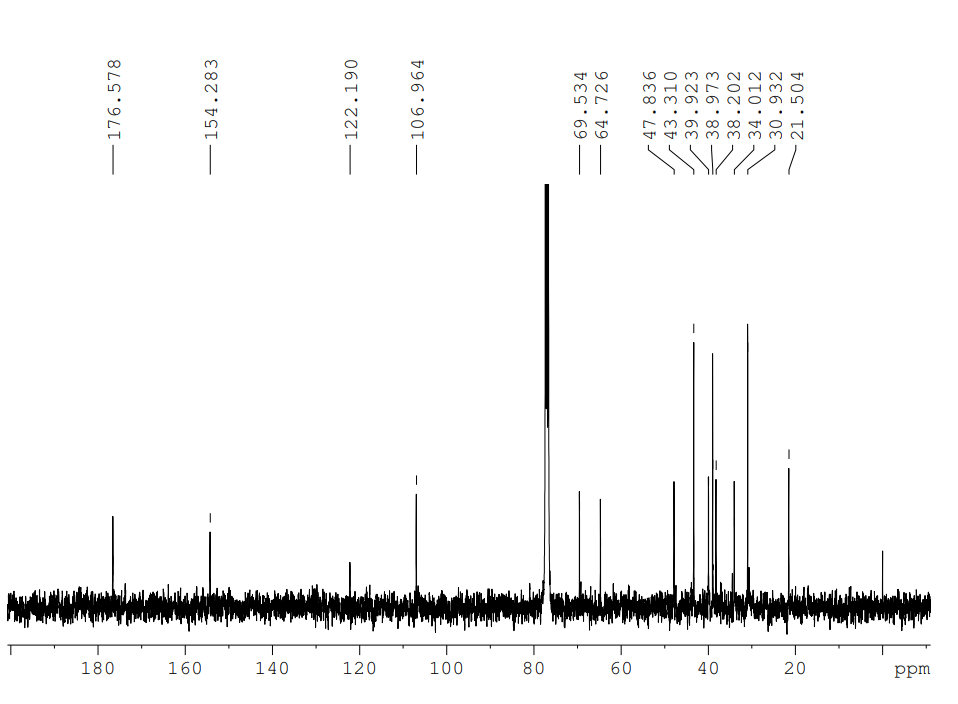


**Figure S2 |** ^13^ C NMR spectrum of unknown impurity (vildagliptin and metformin tablet)


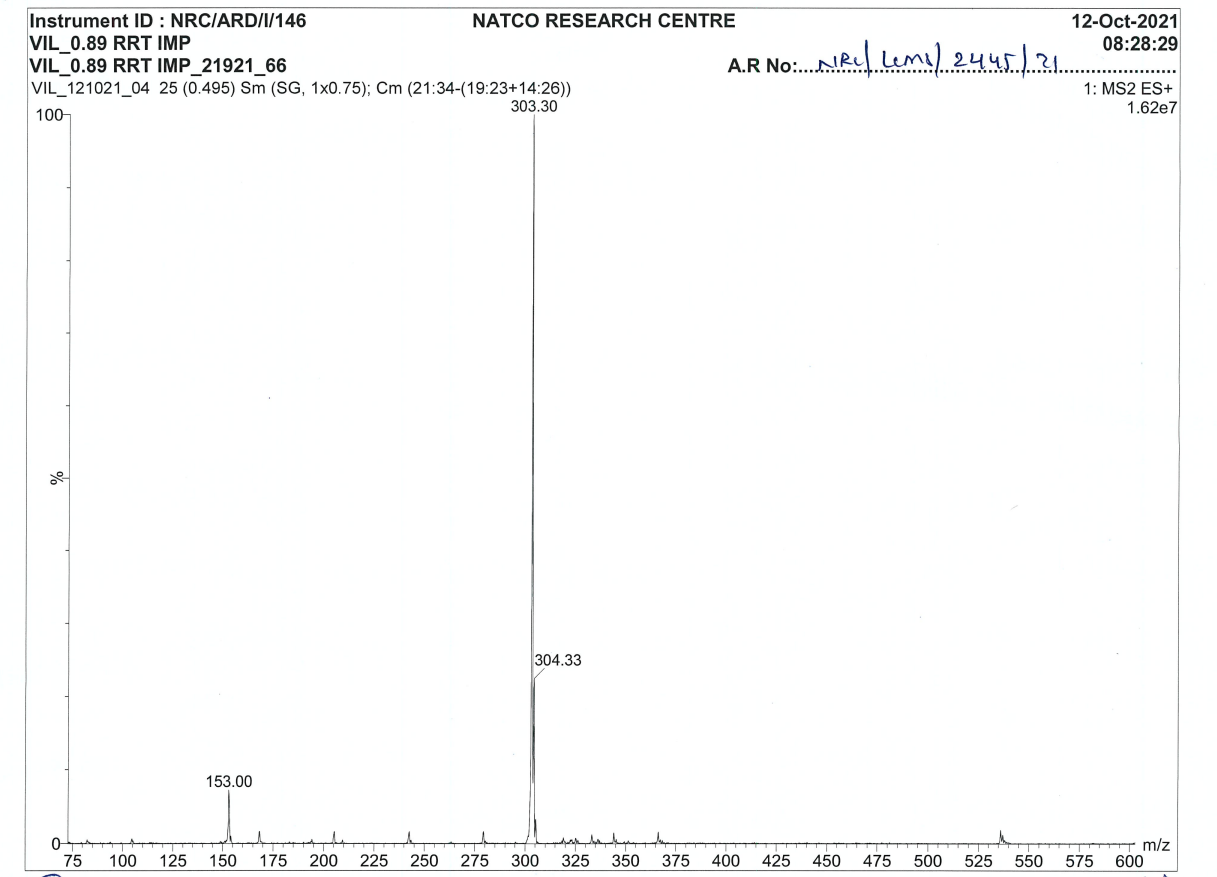


**Figure S3 |** Mass spectrum of unknown impurity (vildagliptin and metformin tablet)


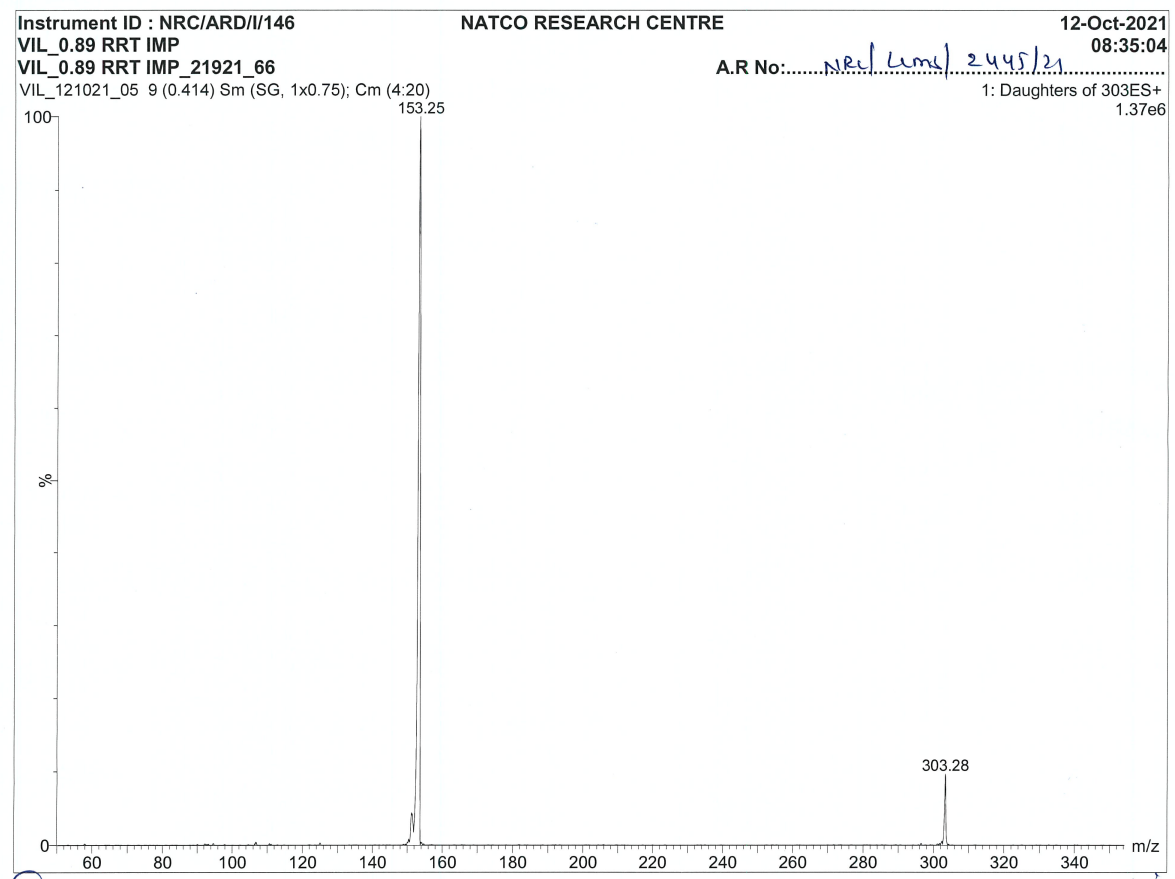


**Figure S4 |** MS/MS spectrum of unknown impurity (vildagliptin and metformin tablet)


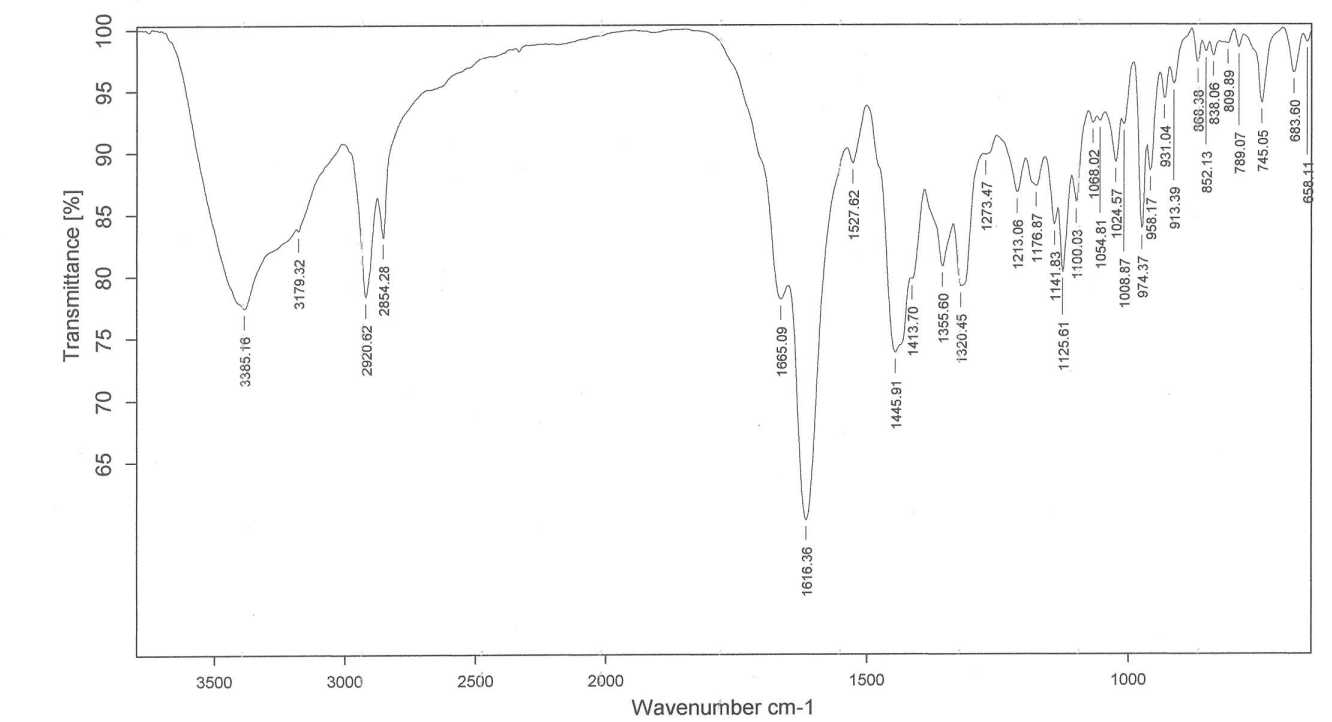


**Figure S5 |** FT-IR spectrum of unknown impurity (Thermal degradation)


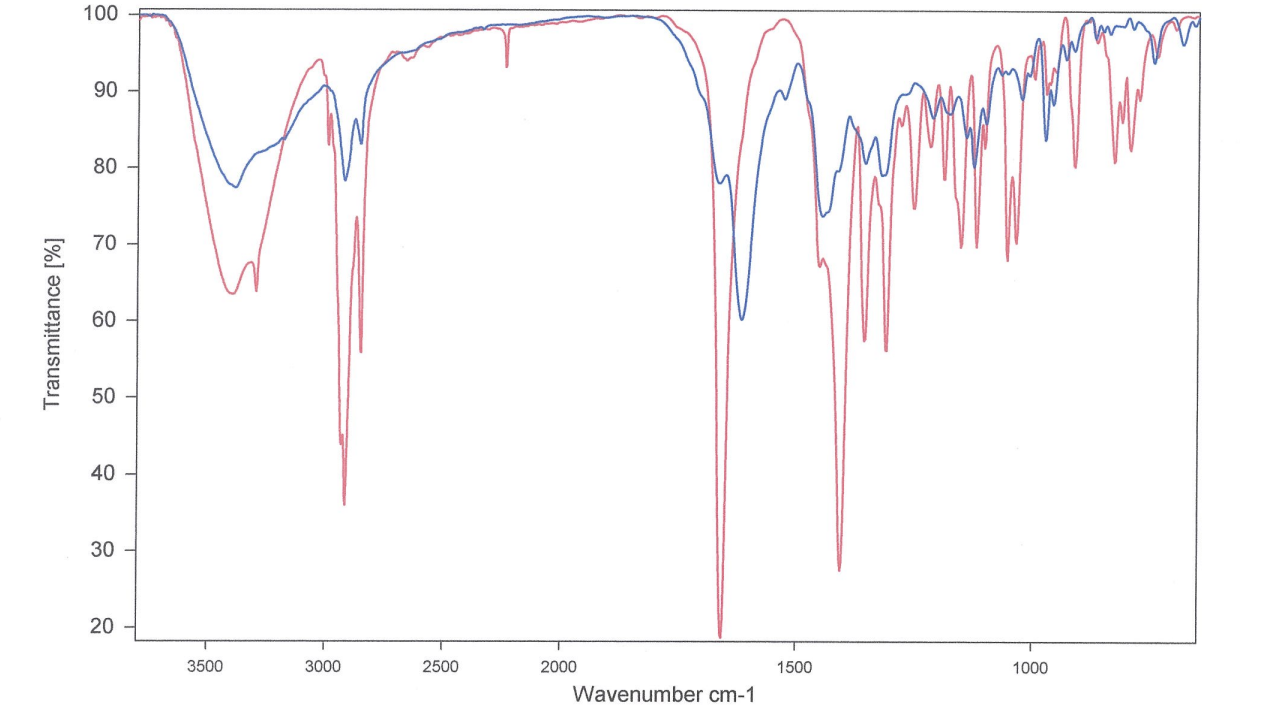


Unknown Impurity

Vildagliptin

**Figure S6 |** FT-IR Overlay spectra of vildagliptin and unknown impurity


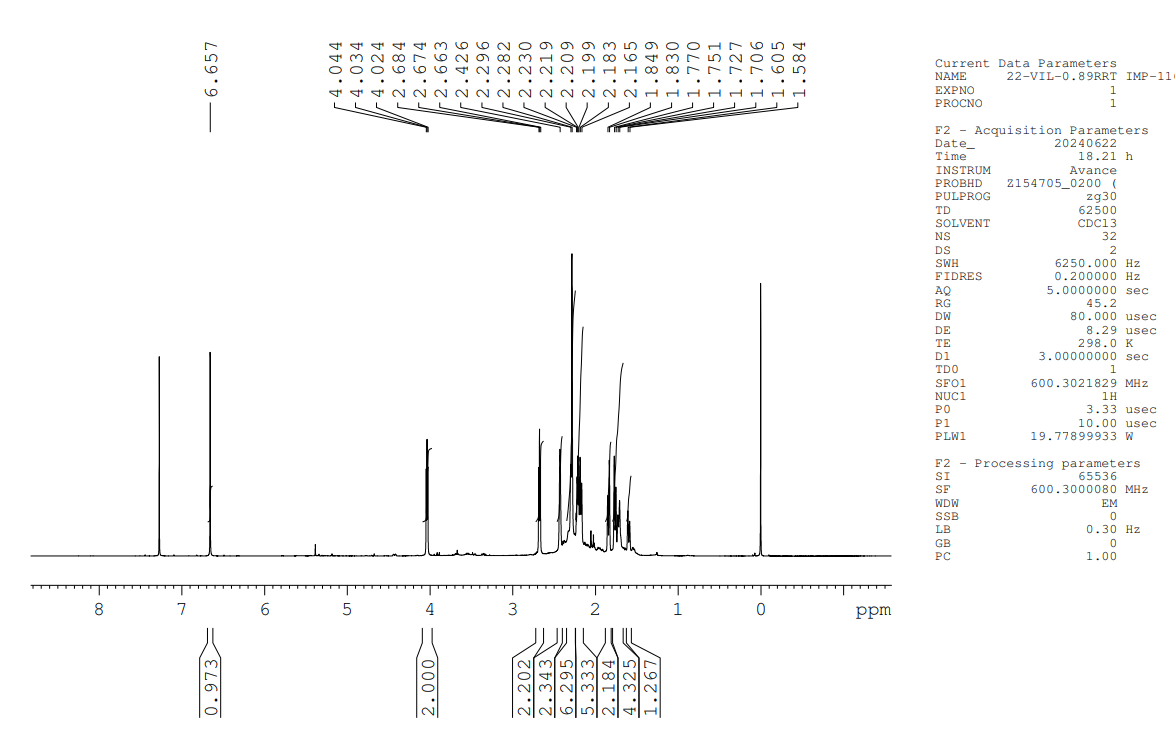


**Figure S7 |** ^1^H NMR spectrum of unknown impurity (Thermal degradation).


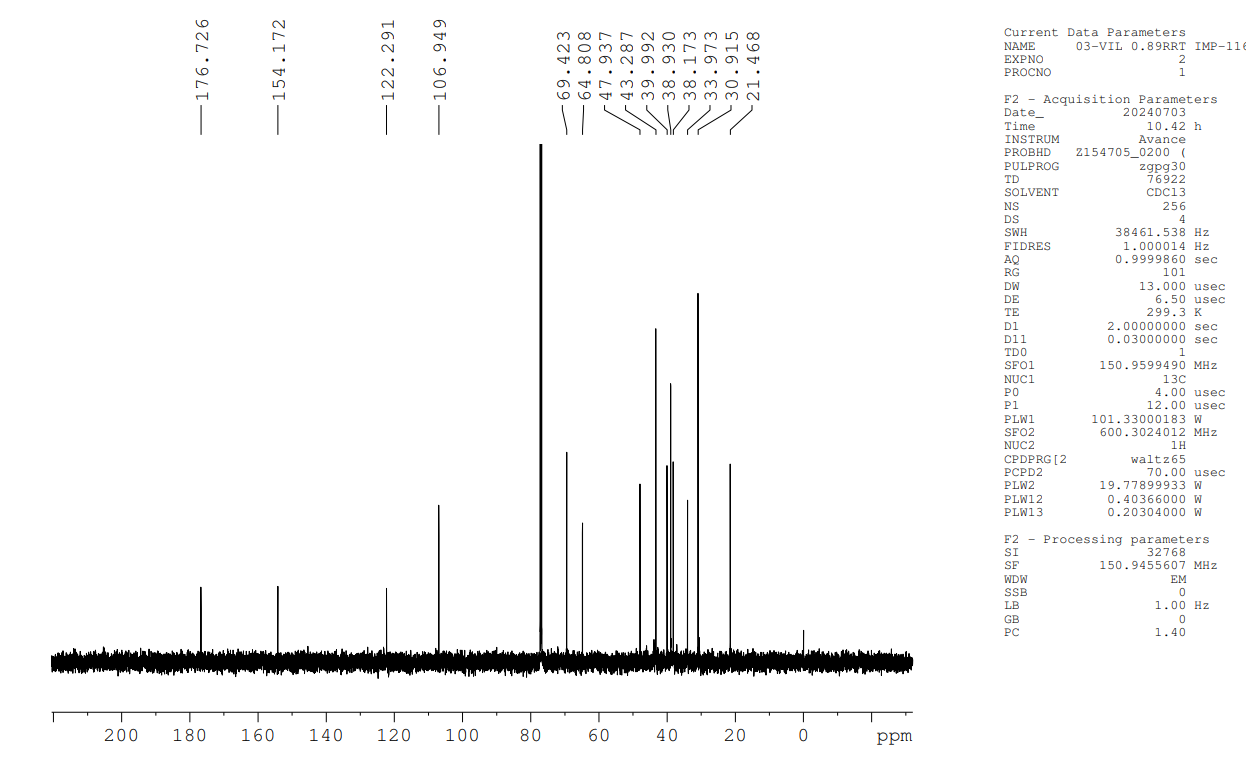


**Figure S8 |** ^13^CNMR spectrum of unknown impurity (Thermal degradation).


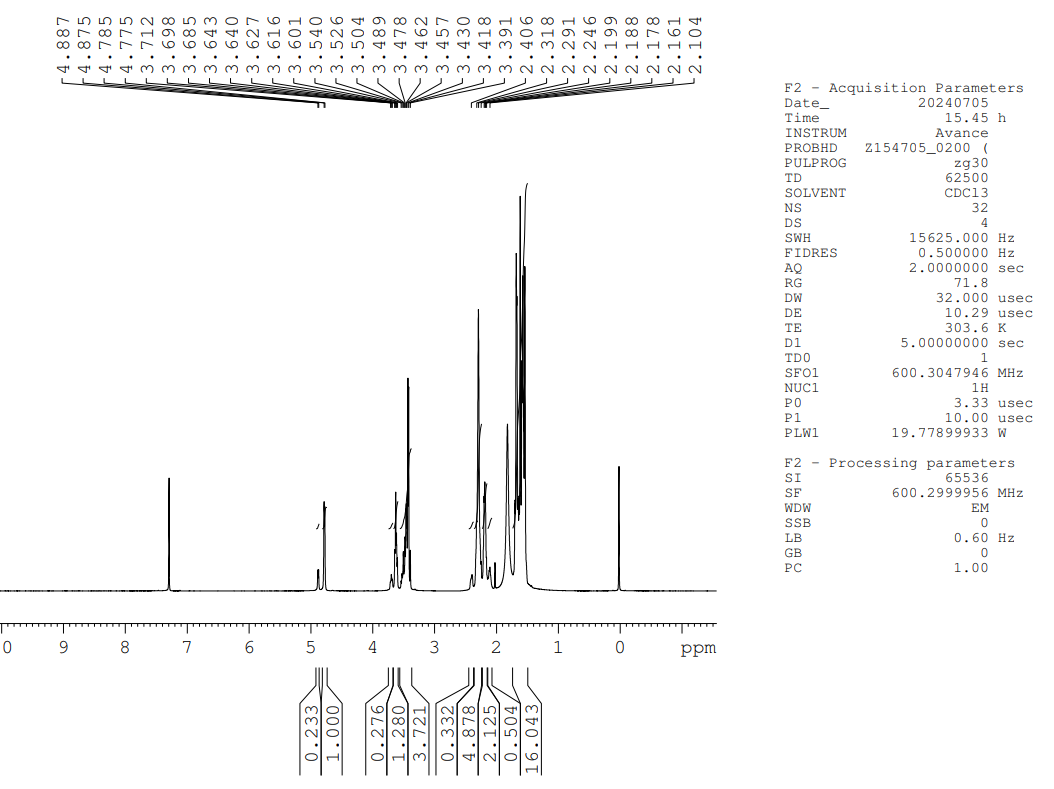


**Figure S9 |** ^1^H NMR spectrum of vildagliptin.


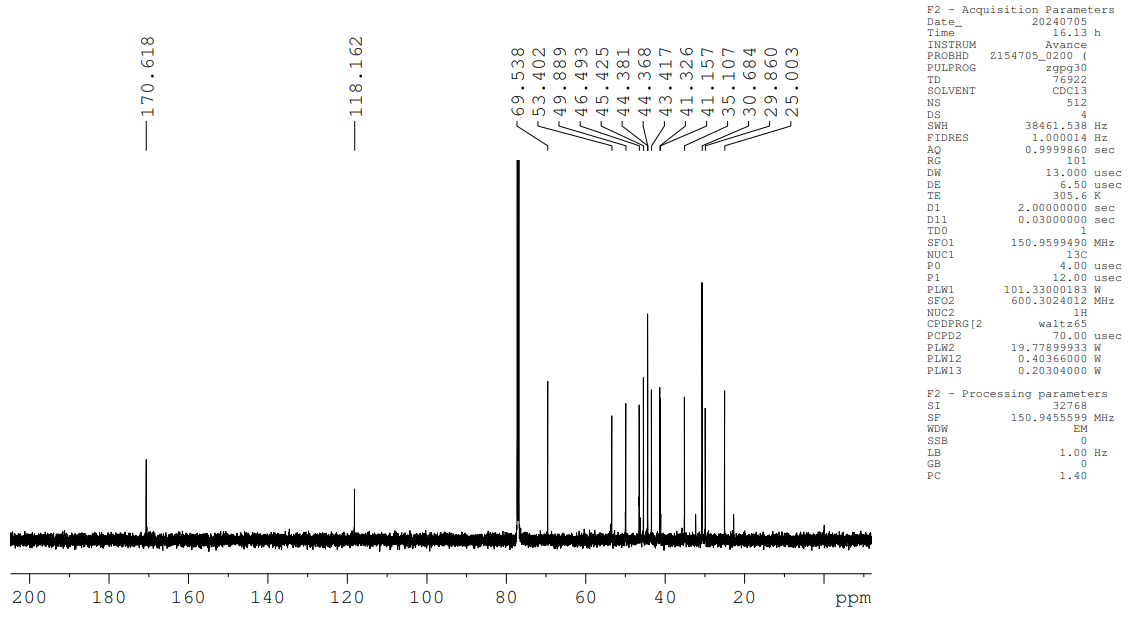


**Figure S10 |** ^13^C NMR spectrum of vildagliptin.


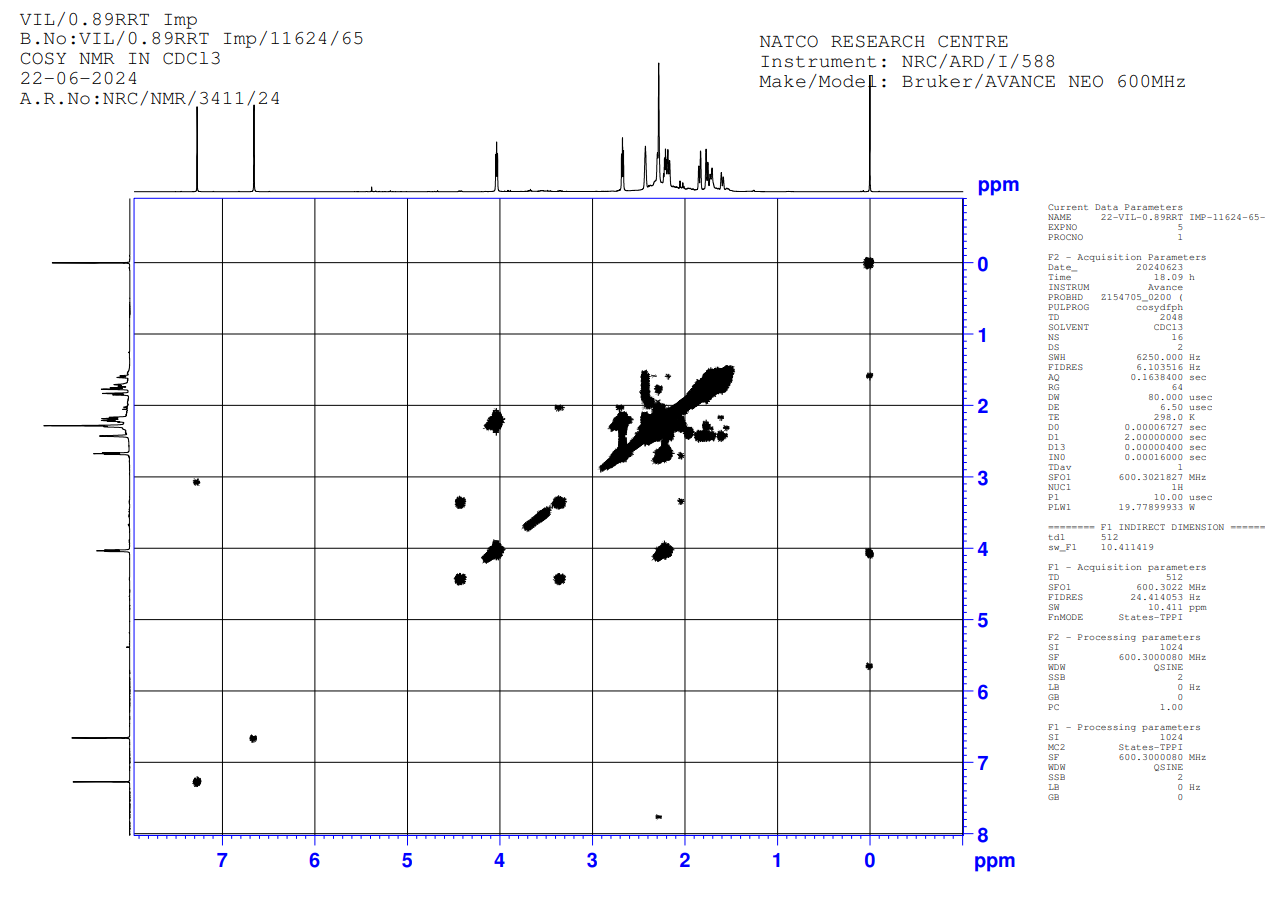


**Figure S11 |** ^1^H-^1^H COSY NMR spectrum of unknown impurity (Thermal degradation).


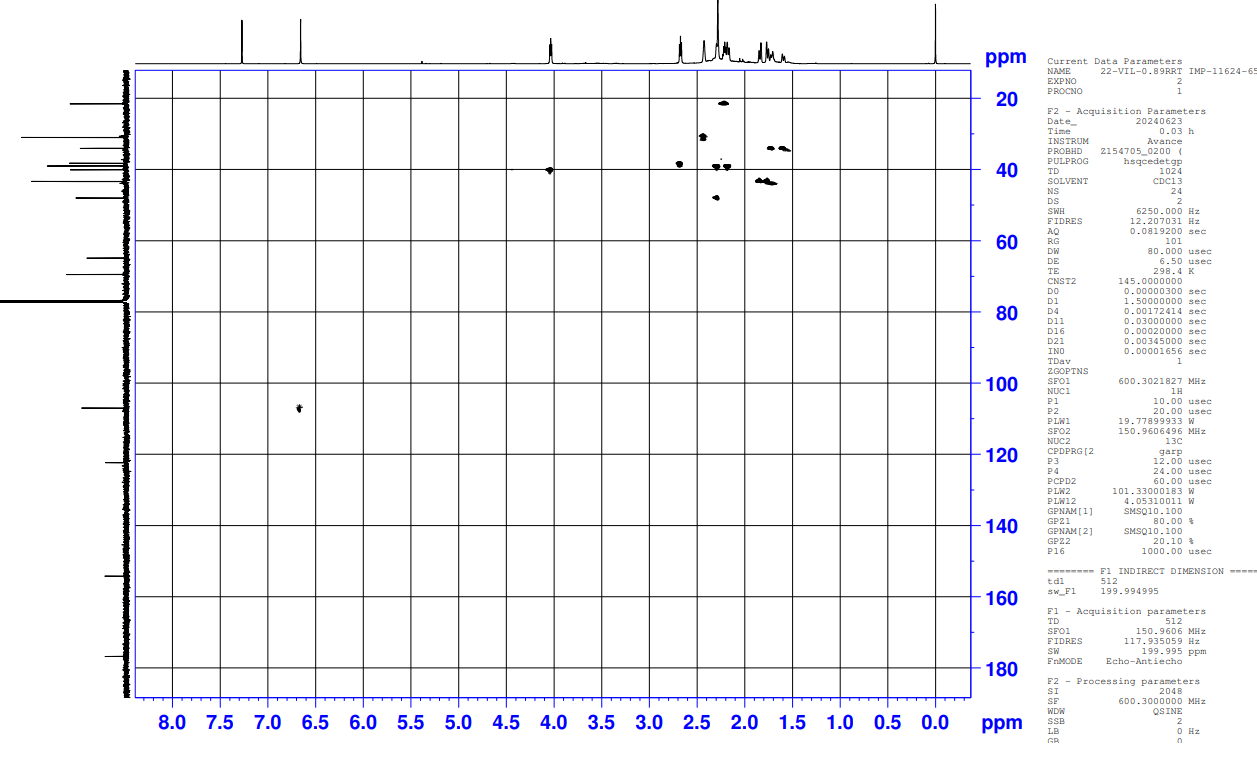


**Figure S12 |** ^1^H-^13^C HSQC NMR spectrum of unknown impurity (Thermal degradation).


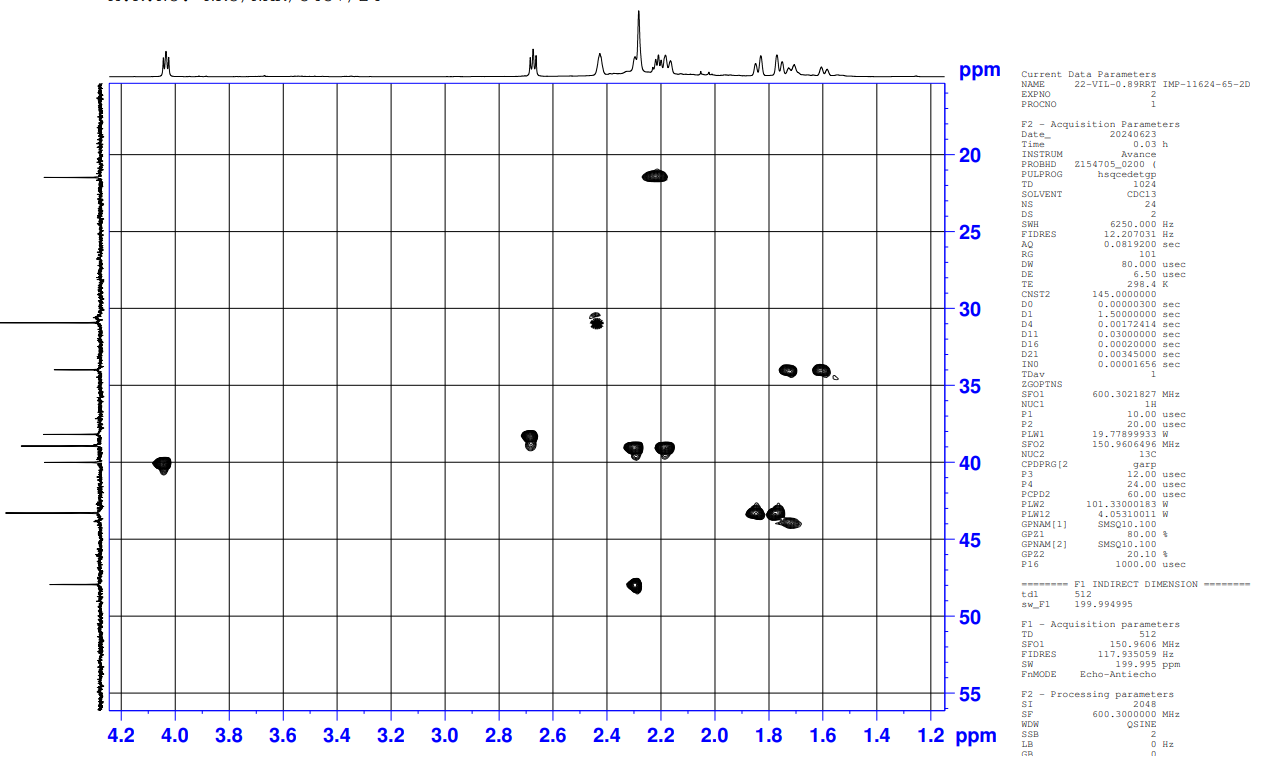


**Figure S13 |** ^1^H-^13^C HSQC (expansion) NMR spectrum of unknown impurity (Thermal degradation).


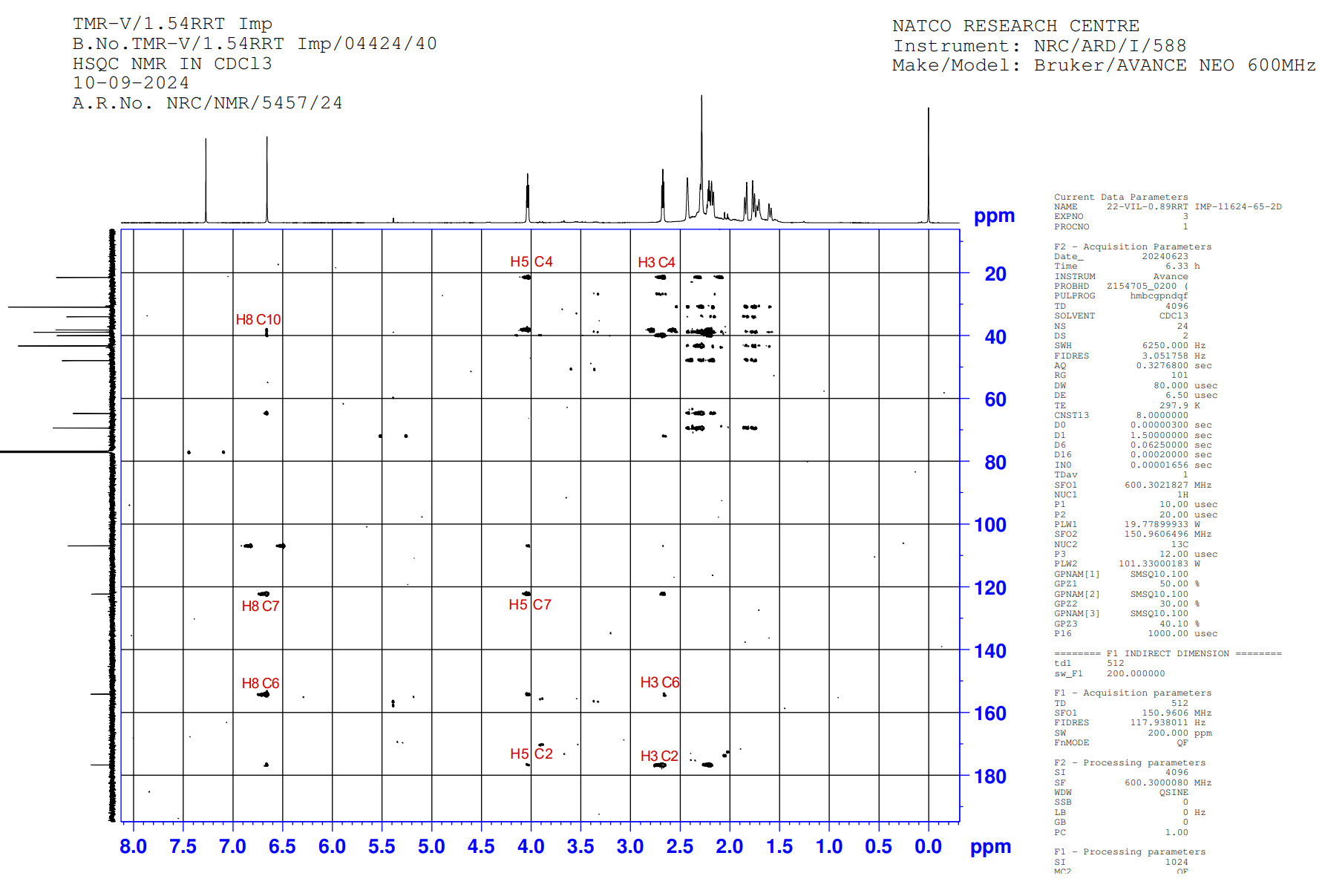


**Figure S14 |** ^1^H-^13^C HMBC NMR spectrum of unknown impurity (Thermal degradation).


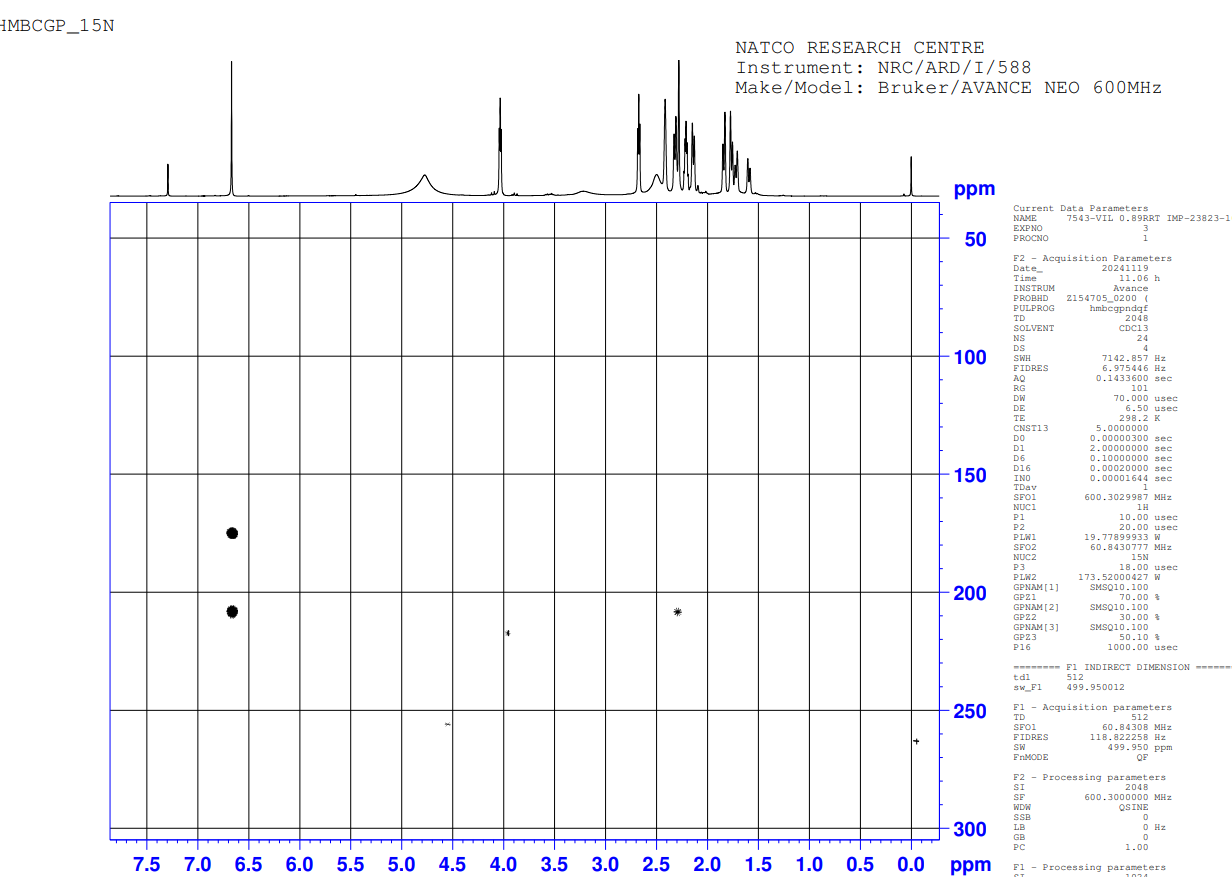

**Figure S15 |** ^1^H-^15^N HMBC (cnst13=5, J(XH) long range) NMR spectrum of unknown impurity (Thermal degradation).


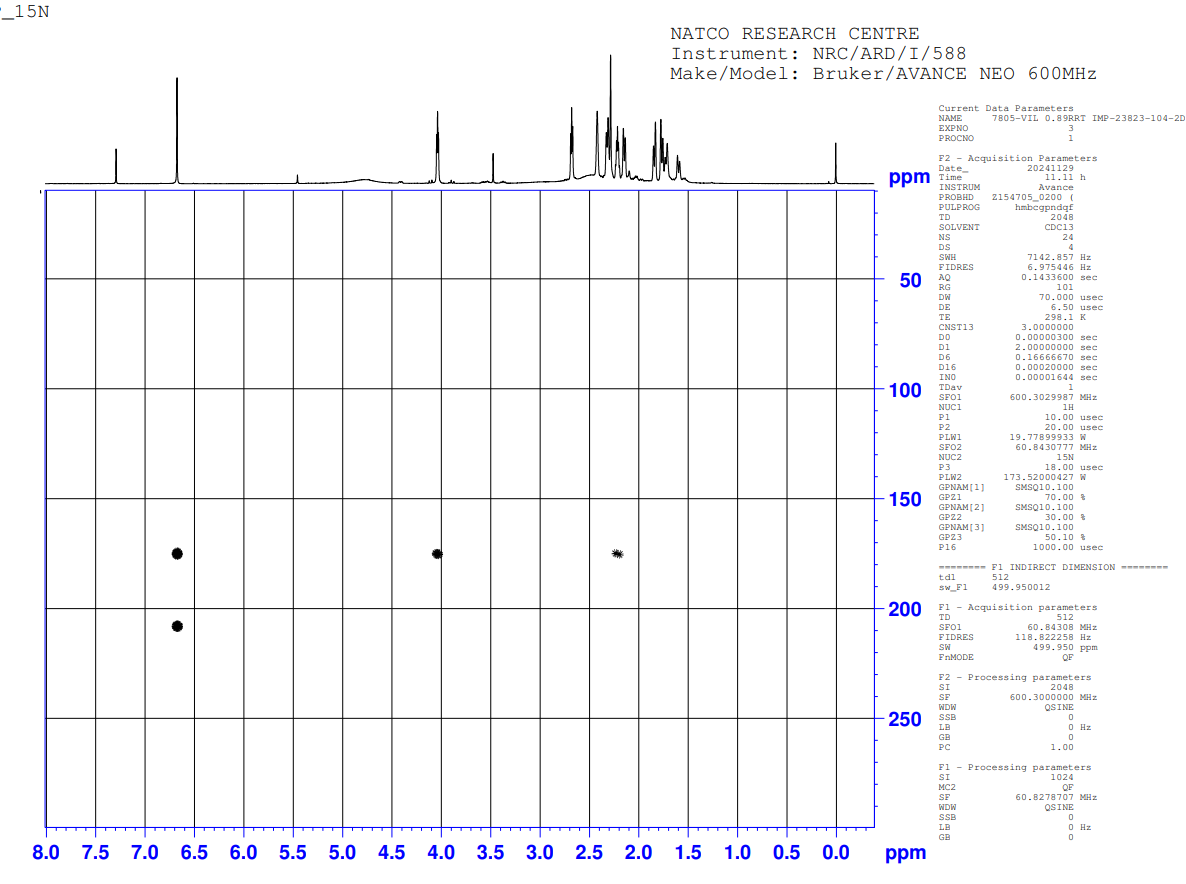

**Figure S16 |** ^1^H-^15^N HMBC (cnst13=3, J(XH) long range) NMR spectrum of unknown impurity (Thermal degradation).


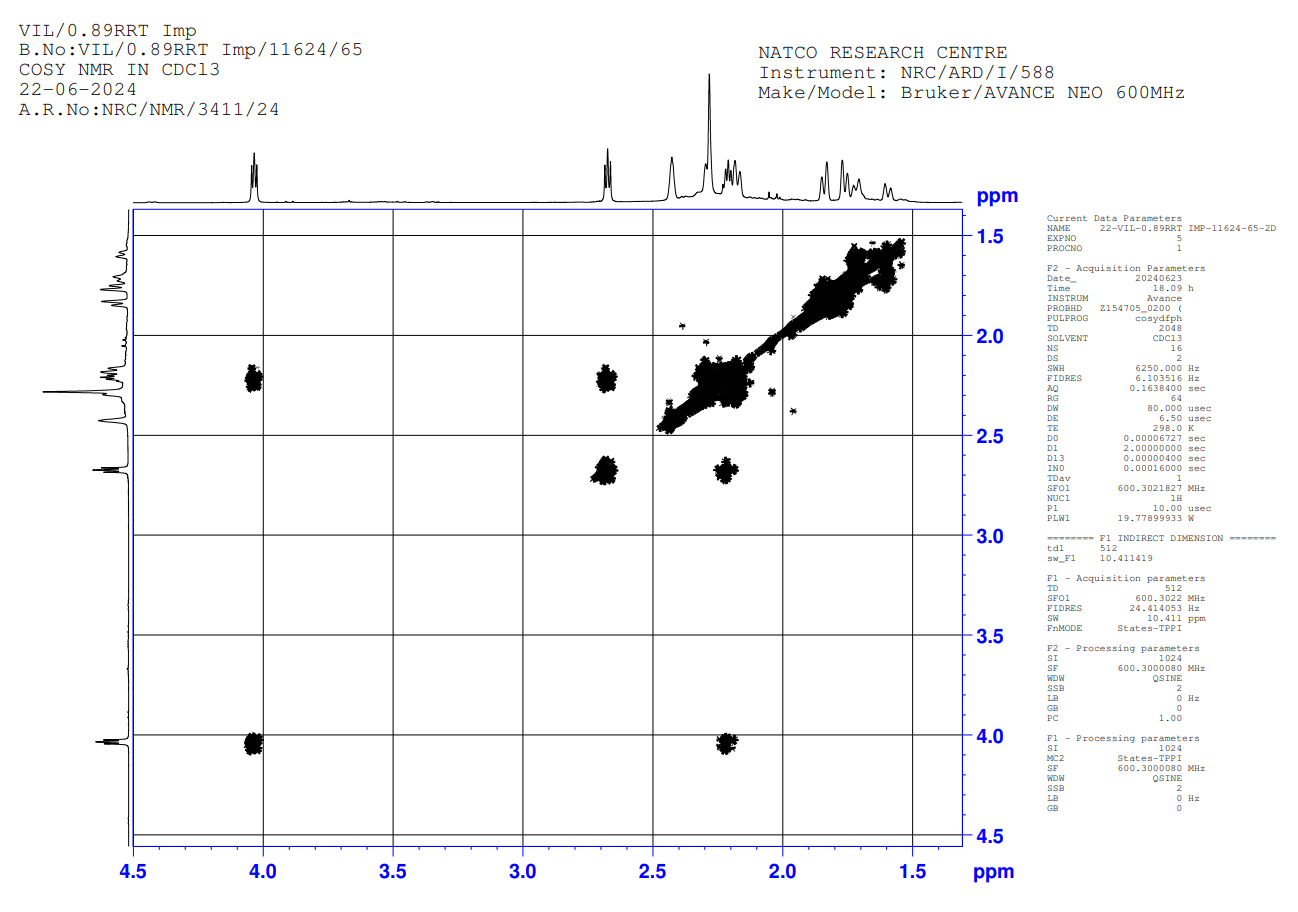


**Figure S17 |** ^1^H-^1^H COSY NMR (expansion) spectrum of unknown impurity (Thermal degradation).


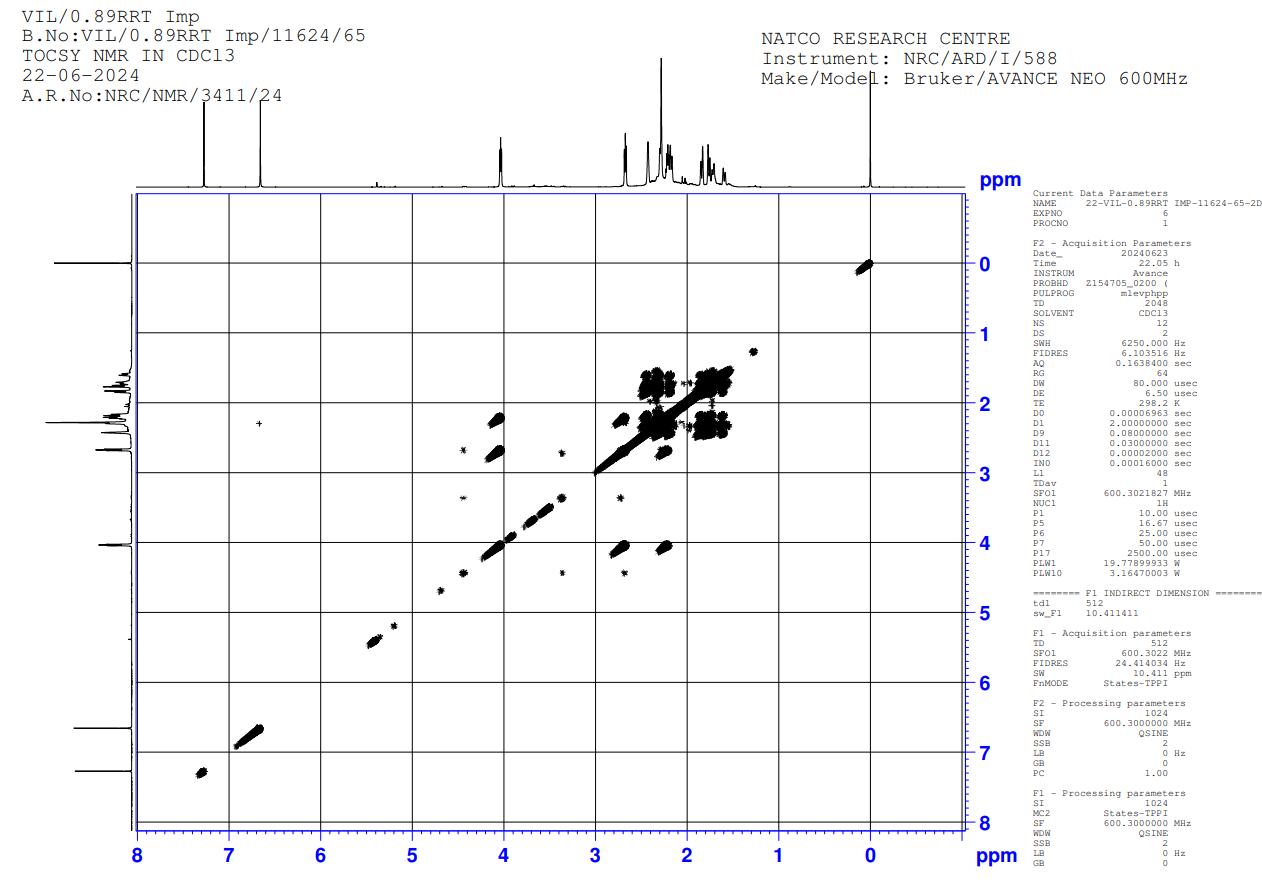


**Figure S18 |** ^1^H-^1^H TCOSY NMR spectrum of unknown impurity (Thermal degradation).


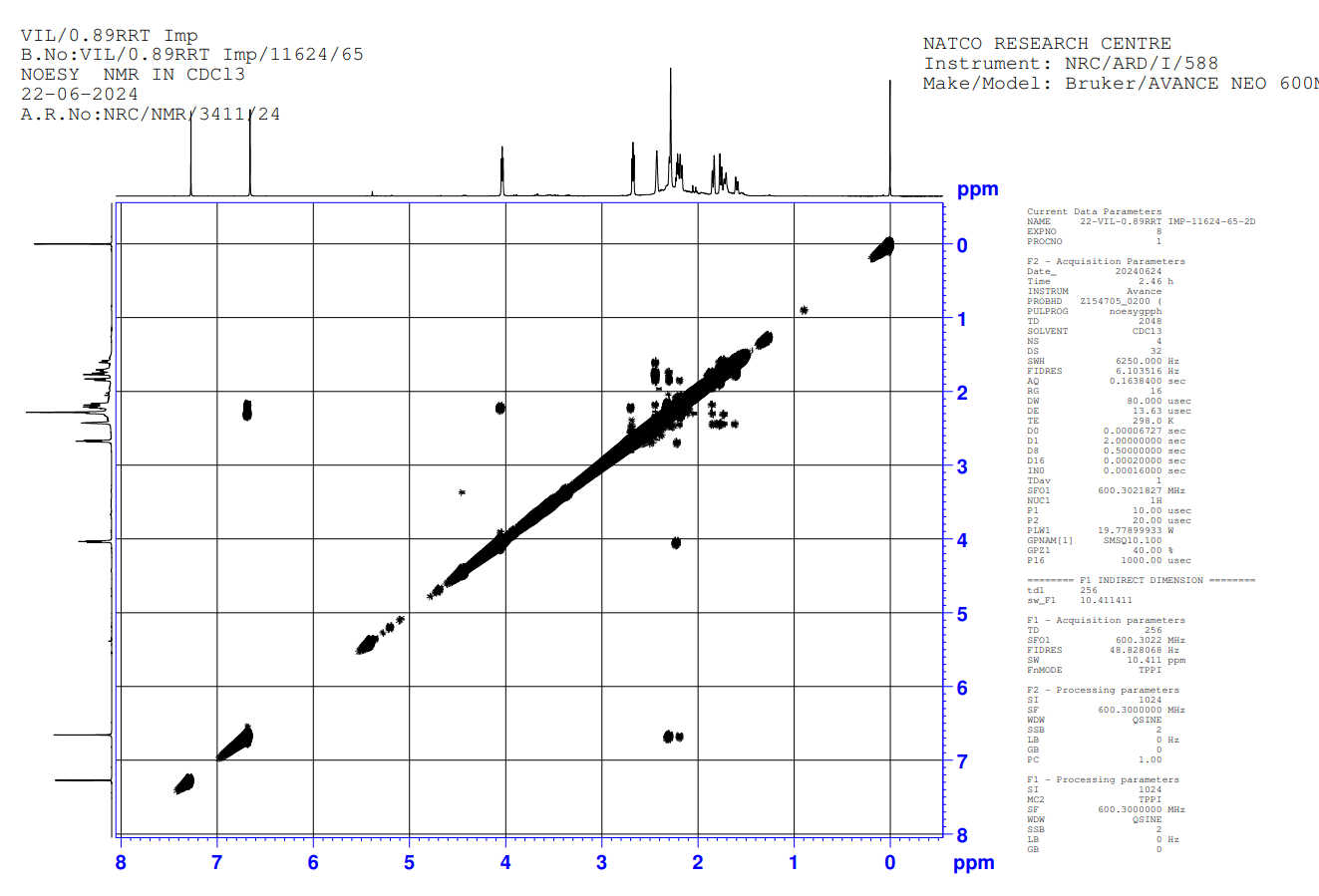


**Figure S19 |** NOESY NMR spectrum of unknown impurity (Thermal degradation).


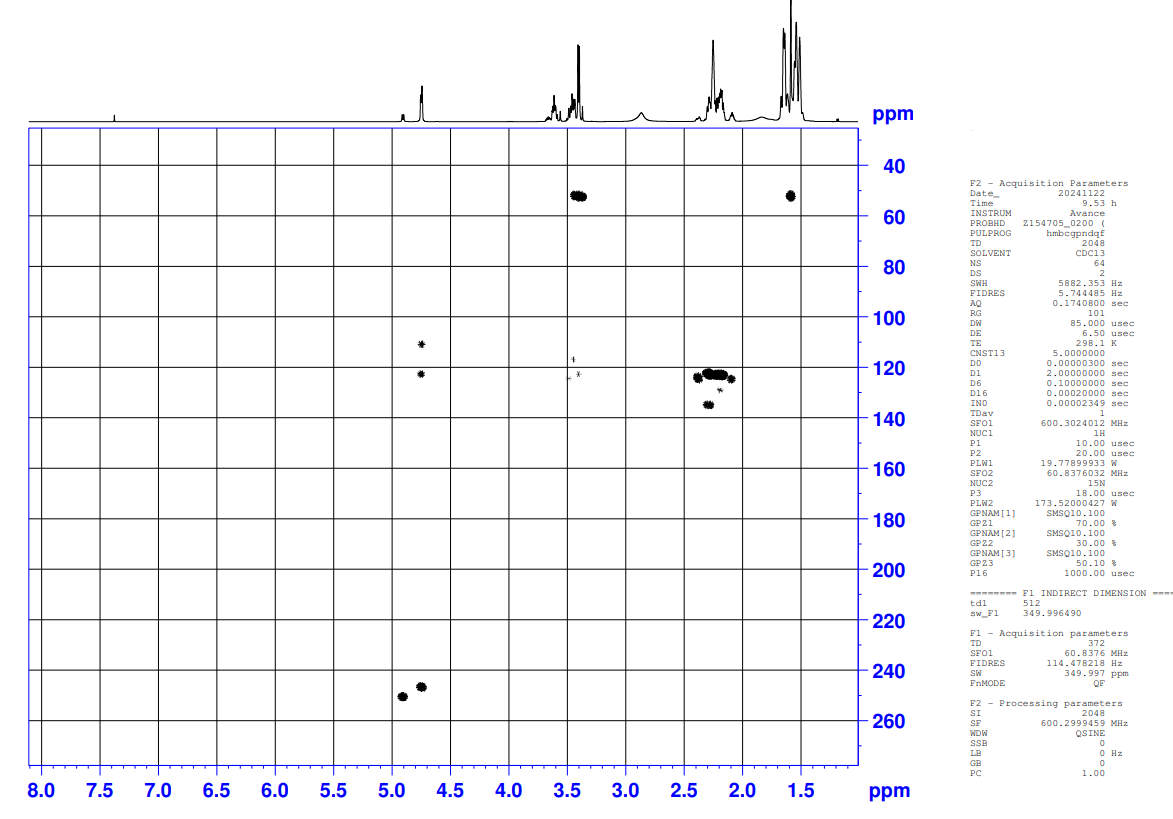


**Figure S20 |** ^1^H-^15^N HMBC NMR spectrum of vildagliptin.


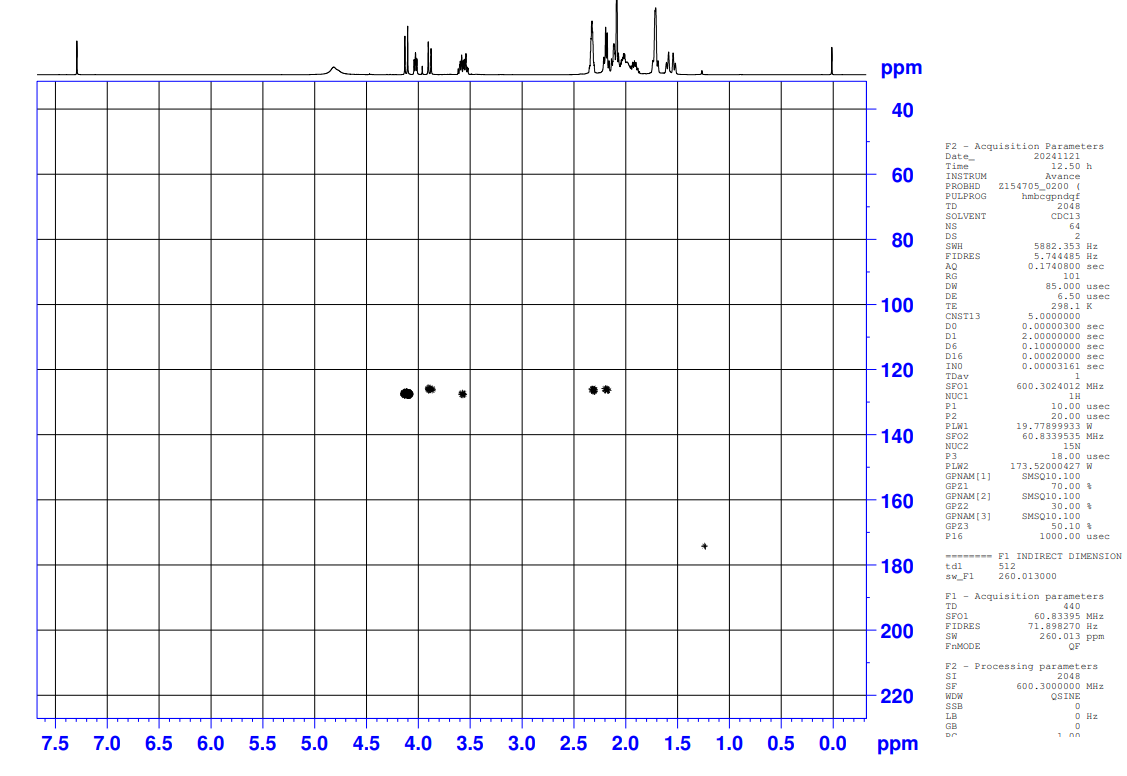


**Figure S21 |** ^1^H-^15^N HMBC NMR spectrum of diketo impurity.

**Vildagliptin diketo impurity**

Exact Mass: 304.1786

Molecular Formula: C_17_H_24_N_2_O_3_

2-(3-hydroxy-1-adamantyl)-6,7,8,8a-tetrahydro-3*H*-pyrrolo[1,2-a]pyrazine-1,4-dione

**Figure S22 |** Structure of vildagliptin diketo impurity.


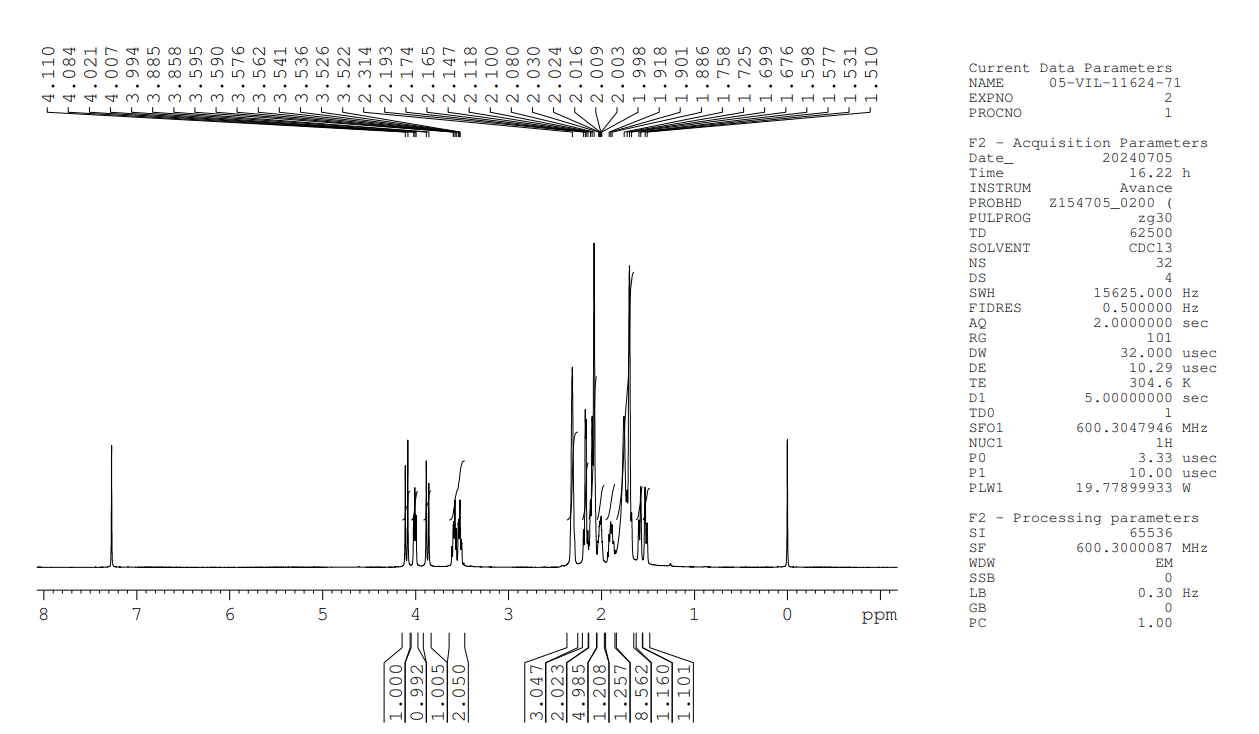


**Figure S23 |** ^1^H NMR spectrum of diketo impurity.


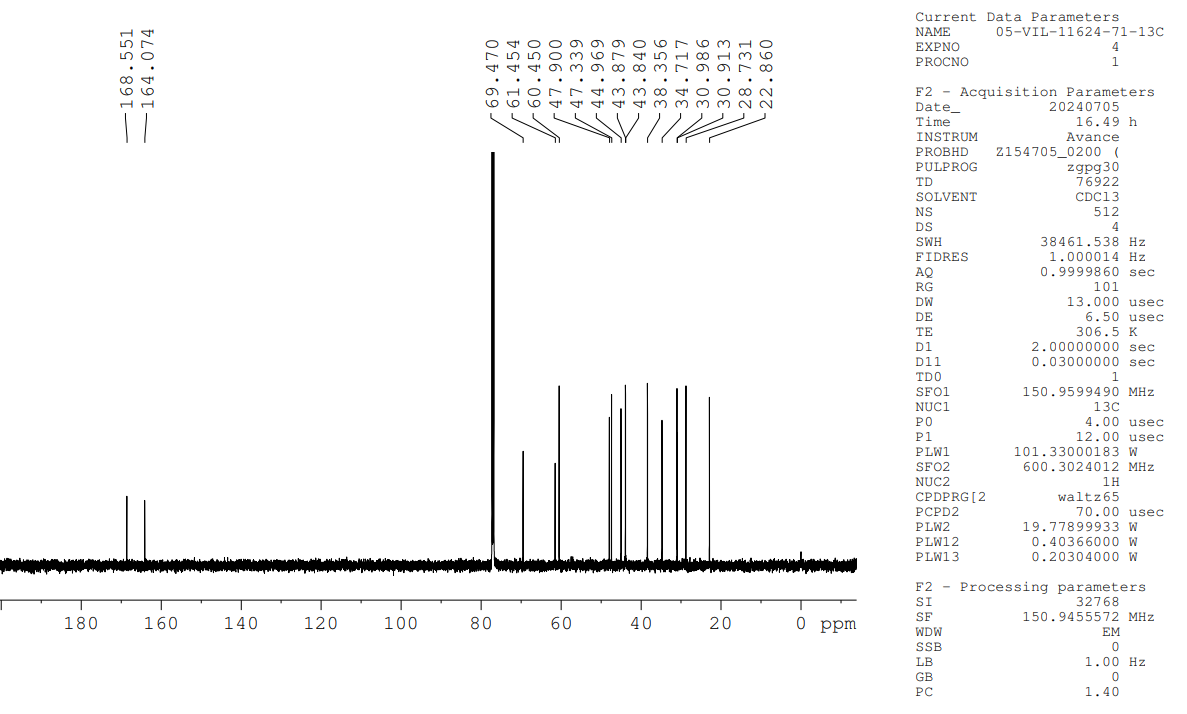


**Figure S24 |** ^13^C NMR spectrum of diketo impurity.


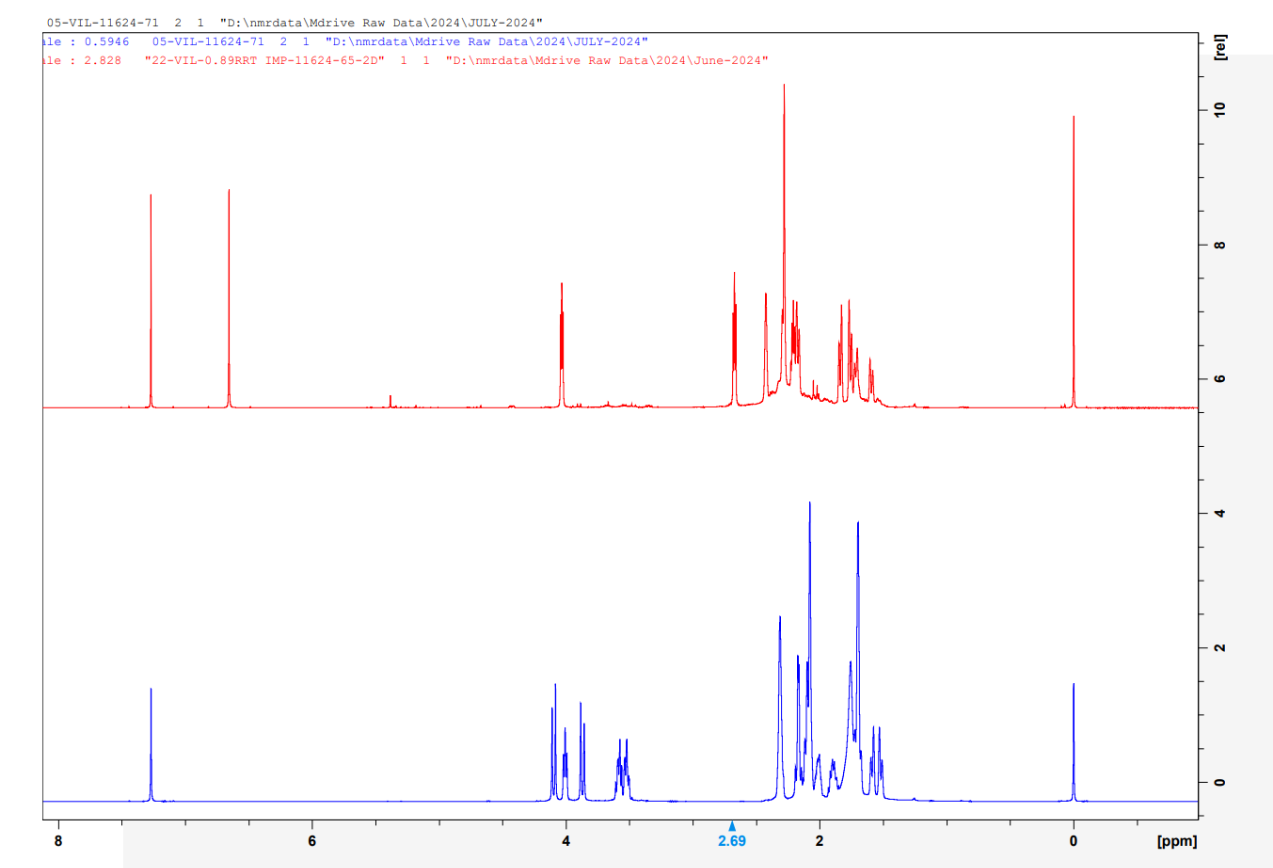


Diketo impurity

Unknown impurity

**Figure S25 |** ^1^H NMR overlay spectra of unknown impurity and diketo impurity.


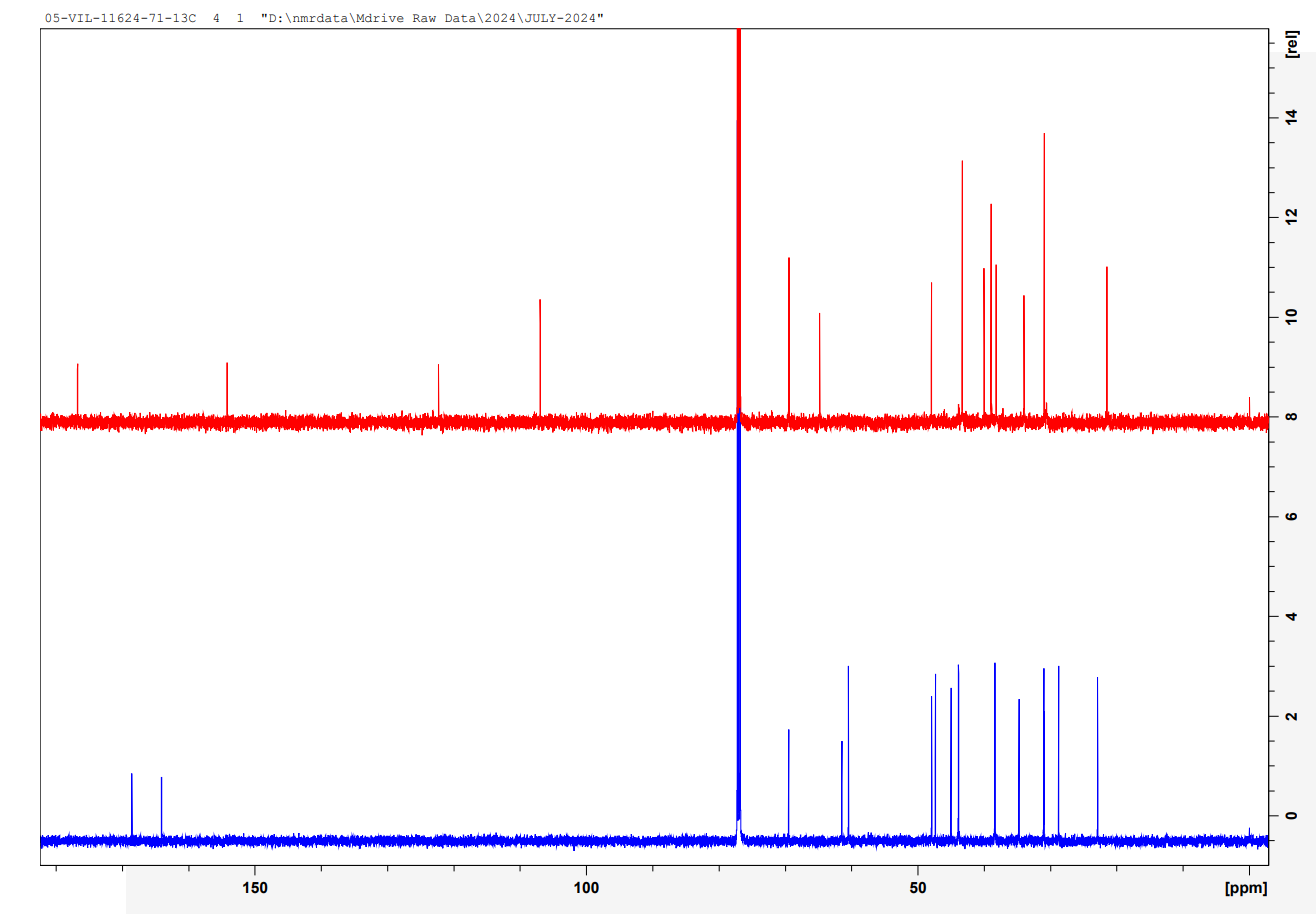
 **Figure S26 |** ^13^C NMR overlay spectra of unknown impurity and diketo impurity.

Diketo impurity

Unknown impurity

**Figure S27 |** Proposed degradation pathway of unknown impurity.
